# *Caenorhabditis elegans *Heterochromatin protein 1 (HPL-2) links developmental plasticity, longevity and lipid metabolism

**DOI:** 10.1186/gb-2011-12-12-r123

**Published:** 2011-12-20

**Authors:** Peter Meister, Sonia Schott, Cécile Bedet, Yu Xiao, Sabine Rohner, Selena Bodennec, Bruno Hudry, Laurent Molin, Florence Solari, Susan M Gasser, Francesca Palladino

**Affiliations:** 1 Friedrich Miescher Institute for Biomedical Research, Maulbeerstrasse 66, 4058 Basel, Switzerland; 2 Cell Fate and Nuclear Organization, Institute of Cell Biology, University of Bern, Baltzerstrasse 6, CH-3012 Bern, Switzerland; 3 Laboratory of Molecular and Cellular Biology, CNRS, Université de Lyon, Ecole Normale Supérieure, 69364 Lyon Cedex 07, France; 4 Development Biology Institute of Marseille Luminy, UMR 6216, Case 907 - Parc Scientifique de Luminy, 13288 Marseille Cedex 9, France; 5 Center of Molecular and Cellular Physiology and Genetics, Université Lyon 1, CNRS UMR 5534, 69622 Villeurbanne, France

## Abstract

**Background:**

Heterochromatin protein 1 (HP1) family proteins have a well-characterized role in heterochromatin packaging and gene regulation. Their function in organismal development, however, is less well understood. Here we used genome-wide expression profiling to assess novel functions of the *Caenorhabditis elegans *HP1 homolog HPL-2 at specific developmental stages.

**Results:**

We show that HPL-2 regulates the expression of germline genes, extracellular matrix components and genes involved in lipid metabolism. Comparison of our expression data with HPL-2 ChIP-on-chip profiles reveals that a significant number of genes up- and down-regulated in the absence of HPL-2 are bound by HPL-2. Germline genes are specifically up-regulated in *hpl-2 *mutants, consistent with the function of HPL-2 as a repressor of ectopic germ cell fate. In addition, microarray results and phenotypic analysis suggest that HPL-2 regulates the dauer developmental decision, a striking example of phenotypic plasticity in which environmental conditions determine developmental fate. HPL-2 acts in dauer at least partly through modulation of *daf-2*/IIS and TGF-β signaling pathways, major determinants of the dauer program. *hpl-2 *mutants also show increased longevity and altered lipid metabolism, hallmarks of the long-lived, stress resistant dauers.

**Conclusions:**

Our results suggest that the worm HP1 homologue HPL-2 may coordinately regulate dauer diapause, longevity and lipid metabolism, three processes dependent on developmental input and environmental conditions. Our findings are of general interest as a paradigm of how chromatin factors can both stabilize development by buffering environmental variation, and guide the organism through remodeling events that require plasticity of cell fate regulation.

## Background

In most organisms, including mammals, environmental and physiological signals are integrated to regulate metabolism, development and life span. In eukaryotes, these processes are often associated with characteristic epigenetic changes brought about by the activity of chromatin-associated proteins and enzymes that influence the transition between chromatin states, thereby influencing transcriptional activity.

Among the more universally conserved epigenetic factors are members of the Heterochromatin protein 1 (HP1) family. These contribute directly to the formation of nuclear heterochromatic domains, including telomeres and centromeres, through an interaction with tri-methylated lysine 9 of histone H3 (H3K9me3) [1,2]. Both H3K9 methylation and HP1 binding at pericentromeric regions play a crucial role in chromosome segregation during mitosis [3,4]. Within euchromatic regions, however, the function of HP1 proteins in the control of gene expression is complex. This involves interactions with other proteins or RNA components, and leads to either gene activation or repression depending on the chromatin context [5-7].

In many cases, the euchromatic functions of HP1 appear to be specific to different homologues within a species, which display dramatic differences with respect to subcellular localization and function. *Drosophila *HP1a acts as positive regulator of transcription by facilitating H3K36 demethylation through an interaction with the dKDM4A demethylase [8], while HP1c interacts with two related transcription factors, WOC and ROW, on active chromatin domains [9]. HP1a also functions in gene activation through association with nascent transcripts [10], while HP1c was found to link the histone chaperone complex FACT to active RNA polymerase II [11]. In mammals, the binding of HP1γ upstream of euchromatic genes is associated with silencing [12], while its association within the coding region of genes was found to affect transcriptional elongation [13]. More recently, HP1γ and H3K9 tri-methylation were also found to be associated with alternative splicing [14]. Although these studies suggest a role for HP1 family members at both heterochromatic and euchromatic sites, HP1's essential role in centromere function has confounded analysis of its role in development of most species. In *Drosophila*, for example, mutations in HP1a are associated with severe chromosome segregation defects due to a perturbation of centromeric heterochromatin [15]. The ensuing early larval lethality obscures other defects in gene regulation in the homozygous mutant, and renders a systematic analysis of HP1a function in fly development very difficult. Nonetheless, this early lethality could be bypassed by the conditional RNA interference (RNAi) inactivation of HP1 in transgenic flies. This resulted in a preferential lethality of males, due to the sex-specific regulation of genes encoding cell cycle regulators [16]. Intriguingly, cell type-specific effects have also been ascribed to the various HP1 homologues in mammals. Both *in vivo *localization and RNAi studies show that they play distinct roles in the differentiation of different cell types [17-20]. The specificity and sub-nuclear distribution of each homologue appears to be influenced by humoral signals and microenvironmental cues [21].

Exploiting the holocentric nature of *Caenorhabditis elegans *chromosomes, we are able to examine the role of HP1 during worm development without having to subvert an essential role in centromere stability. We have previously shown that deletion of the *C. elegans hpl-2 *gene, which encodes an HP1 homologue, results in temperature-sensitive non-lethal developmental abnormalities, including defects in vulval cell-fate specification, and desilencing of repetitive arrays in the germline [22,23]. Consistent with the holocentric nature of *C. elegans *chromosomes, no defects in chromosome segregation were observed in *hpl-2 *loss-of-function mutants [22,24,25]. However, in vulval cell-fate specification, HPL-2 appeared to act through repression of specific genes, including *lin-3*/EGF inducer and *lin-39*/HOX [26]. In the present study, expression microarray analysis was performed using *hpl-2 *null mutants at embryonic and larval developmental stages. At the L3 larval stage, when the germline develops, germline-enriched genes are found to be overexpressed in *hpl-2 *mutant animals. This is consistent with a role for HPL-2 in preventing ectopic expression of germline genes in the soma [27,28], and/or a repressive function in the germline [22]. Intriguingly, transcripts that are down-regulated in the absence of *hpl-2 *at the larval stage stem from genes involved in extracellular matrix-related functions and in lipid metabolism and transport. Comparison of our expression data with HPL-2 chromatin immunoprecipitation (ChIP)-on-chip profiles available through the modENCODE Consortium [29] shows that several of these genes are bound by HPL-2, suggesting a regulatory function.

This analysis has revealed a significant overlap between HPL-2-regulated genes and genes whose expression is altered in dauer diapause, an alternative developmental program that worms undergo under stressful conditions. We find that *hpl-2 *interacts genetically with both the transforming growth factor (TGF)-β and the insulin/insulin-like growth factor-I-like receptor (IIR) signaling pathways, which are the major determinants of dauer formation [30]. Moreover, the dauer exit process can be restored by expressing HPL-2 in either neuronal or intestinal cells. Altogether, this suggests that HPL-2 regulates both the decision to enter and exit this developmental stage, as well as controlling the tissue remodeling that accompanies the dauer state.

We are able to attribute additional phenotypes related to dauer to HPL-2 function. Specifically, *hpl-2 *mutants show an increased lifespan that is dependent on both *daf-2 *and the downstream transcription factor *daf-16*/FOXO, mimicking dauer-constitutive mutants of the *daf-2*/IIR pathway. At the same time, *hpl-2 *mutant animals show significant alterations in specific aspects of lipid metabolism, mediated at least in part through the regulation of key phospholipid metabolism enzymes. We suggest that these in turn influence lifespan through the regulation of lipid homeostasis. Together, our data suggest that rather than globally affecting the expression of a large number of genes, HPL-2 specifically controls genes whose expression impinges on the highly intertwined processes of dauer development, longevity and lipid metabolism.

## Results

### Overview of stage-specific transcriptional responses

Previous studies have suggested biological roles for HPL-2 at larval stages of *C. elegans *growth, while no obvious defects in embryonic development were observed in its absence [24]. To detect changes relevant for the *hpl-2 *phenotypes, we carried out DNA microarray experiments comparing expression profiles of wild-type and *hpl-2 *loss-of-function worms grown at 20°C and synchronized at the embryonic or L3 larval stages.

**Figure 1:**
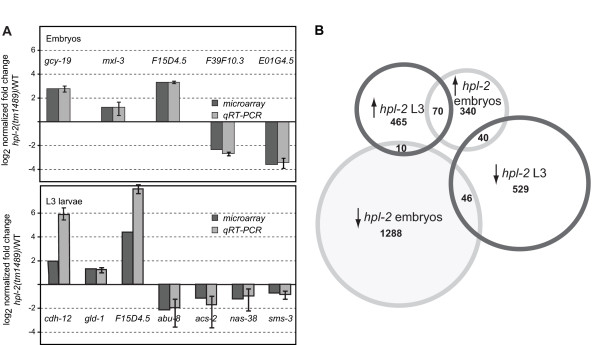
**Validation of microarray results and overlap between embryonic and larval gene sets**. **(a) **Correlation between results obtained by microarray and quantitative RT-PCR (qRT-PCR) for selected genes in embryos and L3 larvae. For qRT-PCR expression values, data are the mean of three independent biological replicas. Error bars indicate standard deviation. *pmp-3 *and *cdc-42 *were used for normalization and the RNA level of each gene of interest was normalized to the mean of both reference genes. **(b) **Venn diagram showing overlap between up- and down-regulated genes in embryonic and L3 larval stages.

To analyze the microarray data, we applied a statistical threshold with a cutoff of *P *< 0.05 (empirical Bayes statistics) and a fold change > 1.5 for mutant versus wild-type samples. The curated list of genes showed a total of 1,794 embryonic transcripts and 1,160 L3 stage transcripts that were misregulated at least 1.5-fold in the *hpl-2 *mutant background compared to wild-type animals (Additional file 1; NCBI [GSE33700]). To further validate the array findings, 12 genes with different developmental expression patterns and different biological functions were selected as targets for RT-PCR (Figure 1a). For all 12 genes, a good correlation between microarray data and quantitative PCR (qPCR) expression values was observed. Of the total number of responsive genes (2,954), only 5.6% were found to be misregulated at both developmental stages (Figure 1b), confirming that HLP-2 has distinct biological roles at different stages of development. Although the vast majority of commonly regulated genes encode proteins of unknown function, a notable exception is *lin-13*, which was up-regulated in both embryos and larvae. LIN-13 is a zinc finger protein required for HPL-2 recruitment to nuclear foci in embryos [23]. This indicates that a complex feedback control exists between HPL-2 and the important lineage factor LIN-13. Analysis of *lin-13 *expression *in vivo *using a green fluorescent protein (GFP) translational fusion revealed strongly increased GFP expression in *hpl-2 *mutant animals compared to wild type, further validating microarray results (Additional file 2).

In embryos, significantly more genes were down-regulated than up-regulated (1,344 and 450, respectively) in the absence of HPL-2, while approximately the same number of genes were found in each class in L3 larvae (615 and 545, respectively; Figure 1b). Although some of the down-regulated genes are likely to be indirect targets, these results suggest that HPL-2 may also play a significant role in gene activation, as reported for certain other HP1 family proteins in both *Drosophila *and mammalian cells [5,11,13,31,32].

To analyze the responsive genes in more depth, they were divided into functional categories based on gene ontologies (GOs; Table 1). In embryos, up-regulated genes are slightly enriched in GO terms associated with lipid metabolic processes (Fisher exact test (FET) *P *= 0.043), while down-regulated genes are enriched in GO terms associated with RNA metabolic processes (FET *P *= 2 × 10^-6^). L3 up-regulated genes are significantly enriched in GO terms associated with meiotic functions (FET *P *= 4 × 10^-6^). The expression of many of these same genes is enriched in the germline (Additional file 3a) and is likely to reflect the role of HPL-2 and other chromatin-associated proteins in the repression of germ cell fate in the soma [28,33]. However, as our microarray analysis was performed on whole animals, some of these genes might also be up-regulated in the germline, where HPL-2 plays a role in germline transgene silencing and the related phenomenon of germline co-suppression [22,34].

**Table 1:** Enrichment for Gene Ontology terms among *hpl-2-*regulated genes

Term	Count	*P*-value	Fold enrichment
**Embryo down-regulated**			
GO:0006396 - RNA processing	23	4.37E-05	2.7
GO:0006796 - phosphate metabolic process	48	0.017	1.4
**Embryo up-regulated**			
GO:0006631 - fatty acid metabolic process	4	0.043	5
**L3 down-regulated**			
GO:0009712 - catechol metabolic process	5	3E-03	8.0
GO:0043043 - peptide biosynthetic process	4	0.02	8.0
GO:0046942 - carboxylic acid transport	5	3E-03	8.0
GO:0006631 - fatty acid metabolic process	3	0.04	5.0
**L3 up-regulated**			
GO:0007126 - meiosis	15	3.21E-06	4.7
GO:0022402 - cell cycle process	19	3.12E-05	3.1
GO:0045132 - meiotic chromosome segregation	12	3.22E-05	4.0

Gene Ontology (GO) terms are from the biological process ontology. GO information was downloaded from DAVID [110]. In some cases, more specific subtypes of the listed parental terms were also significantly enriched.

The genes that are down-regulated in L3 larva by loss of HPL-2 activity are enriched in GO terms associated with alcohol metabolic processes (FET *P *= 0,003), transport (FET *P *= 0,003), and lipid metabolism (FET *P *= 0,004). Intriguingly, visual inspection of the data revealed that four classes of genes (*abu*, *nas*, *fipr*, *nspb*) that are specifically down-regulated at this larval stage are tightly grouped together in mountain 29 of the *C. elegans *three-dimensional topographical expression map [35] (Additional file 3c). These genes may, therefore, be coordinately regulated by HPL-2. Because all of the phenotypes associated with *hpl-2 *loss of function concern post-embryonic development, we specifically focused our attention on genes showing altered expression at the L3 stage, as we considered these genes as those most likely to be directly responsible for stage-specific phenotypes.

**Figure 2:**
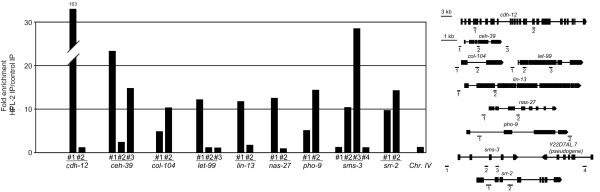
**HPL-2 binding to target genes**. Worm extracts from synchronized L3 worms were subjected to immunoprecipitation (IP) using either HPL-2-specific antibodies or pre-immune serum as control. For each gene tested, a cartoon shows the position of the primers used for quantitative PCR (qPCR) analysis and a histogram representing the fold-enrichment, calculated as the ratio between signal from the antibodies and signal from pre-immune serum, from chromatin immunoprecipitation (ChIP) experiments along the gene. chIV is a primer from an intergenic region on chromosome IV used as an internal control. Similar results were obtained for two to three independent experiments.

Comparison of our expression data with HPL-2 ChIP-on-chip profiles obtained in wild-type L3 larvae (available through the modENCODE Consortium [29]) revealed that a significant number of genes up- and down-regulated in the absence of HPL-2 are bound by HPL-2 (Additional file 4; up-regulated genes FET = 6.7 × 10^-8^; down-regulated genes FET = 1.2 × 10^-4^). ChIP-qPCR analysis confirmed HPL-2 enrichment on a subset of these genes (Figure 2). HPL-2 binding was found both within promoter and coding regions. No correlation was found between promoter or internal binding and either repression or activation of the bound gene in the absence of HPL-2. Therefore, HPL-2 may regulate gene expression by more than one mechanism and in a context-dependent manner, as observed for other HP1 family proteins [7,14,36].

In most organisms HP1 binds methylated H3K9 [1,2]. This interaction appears to be conserved in *C. elegans*, as heterochromatin-like transgenic arrays enriched in methylated H3K9 require HPL-2 for repression [22,37,38]. ChIP analysis confirmed that both H3K9me3 and HPL-2 are enriched on these arrays (Additional file 5). We therefore compared our expression data with ChIP-on-chip profiles of H3K9me1/2/3 in wild-type L3 larvae, also available through the modENCODE Consortium [29]. We reasoned that this approach might help identify genes whose expression is directly regulated by the binding of HPL-2 to methylated H3K9. For each gene list, we calculated the median H3K9me1/2/3 signal over the gene body or the promoter and compared this value to the values obtained for expression-level matched genes. We then plotted the distribution of these median signals as box-whisker representation. In this representation, the bar indicates the median for distribution over genes and the upper and lower edges of the box represent the 25th and 75th percentile, respectively. For the genes that require HPL-2 for full repression, we observe a significantly higher signal for H3K9 mono- and di-methylation, and to a lesser extent tri-methylation, compared to expression-matched promoters and gene bodies from wild-type cells (Additional file 6; Wilcoxon test *P *= 10^-89^, 10^-83^, 10^-34^, respectively, for promoters, 10^-44^, 10^-39 ^and 10^-18^, respectively, for gene bodies). This suggests that at least part of the derepression in *hpl-2 *mutant larvae is indeed direct, that is, due to HPL-2 binding to methylated H3K9. For genes that show lower expression levels in the *hpl*-*2 *mutant, the correlation with methylated H3K9 was much weaker, suggesting indirect effects on gene expression, or possibly the existence of alternative mechanisms for recruiting HPL-2 to euchromatic sites. One such mechanism may be through the LIN-13 zinc finger protein [23].

### *hpl-2 *regulates a subset of dauer genes

**Figure 3:**
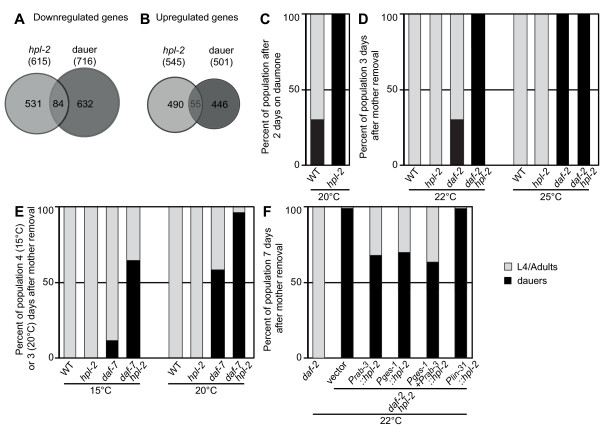
***hpl-2 *regulates dauer diapause**. **(a, b) ***hpl-2 *regulates a subset of dauer-regulated genes. Venn diagrams show the intersection between *hpl-2*-regulated genes identified in this study and dauer-regulated genes [39]. **(c) ***hpl-2 *mutants show an increased response to dauer pheromone. Crude pheromone extract [107] was added to plates at a concentration of 30 μl/ml. Data are the average of triplicate samples, each started with 40 to 50 eggs laid *in situ *at 20°C by three gravid adults that were subsequently removed. Plates were shifted to 25°C and scored for dauer formation after 48 hours. **(d, e) ***hpl-2 *enhances the dauer phenotype of mutants in the *daf-2*/IIS and TGF-β pathways. Synchronized populations of worms were obtained by letting single hermaphrodites lay eggs at 15°C. The mother was then removed and plates shifted to the indicated temperature for development. Dauers were scored 3 days after the removal of the mother for experiments at 20°C, 22°C and 25°C, and 4 days after the removal of the mother for experiments at 15°C. Data are the average of at least three independent experiments. **(f) **Tissue-specific rescue of dauer arrest phenotype of *hpl-2;daf-2 *animals. *hpl-2*;*daf-2(e1370) ***t**ransgenic animals expressing the *hpl-2 *cDNA under control of the indicated tissue-specific promoters were scored for ability to recover from dauer arrest as detailed in Materials and methods. Data are the average of at least three independent experiments with three independent lines for each construct.

Visual inspection revealed that both up- and down-regulated transcripts in *hpl-2 *mutant animals show a significant overlap with genes regulated in animals undergoing dauer arrest [39] (Figure 3a, b; FET *P *< 2 × 10^-6^). In a favorable environment, *C. elegans *develops into an adult through four larval stages (L1 to L4), but in response to starvation or overcrowding, larvae arrest development at the second molt (L2) to form dauer larvae. Dauer animals are non-feeding and stress-resistant, and can remain in this state for months until environmental conditions become favorable again. Dauer formation has been widely used as a model to study the regulatory mechanisms governing morphological change during organismal development [40]. The list of genes whose expression changes during dauer arrest was obtained by comparing expression profiles of wild-type larvae at the L2/L3 larval stage transition to animals undergoing dauer larva formation (pre-dauer L2d/dauer). Our data were obtained from a very similar developmental stage (early L3), which makes the observed overlap particularly relevant. Significantly, we also observed overlap between genes up-regulated during dauer exit [41] and genes down-regulated in the absence of *hpl-2 *(FET *P *= 2 × 10^-16^; Additional file 3b).

The commonly regulated genes fall into four classes. The first class includes genes encoding collagens and metalloproteases involved in the synthesis and degradation of the extracellular matrix and cuticle physiology [39,42,43]. The second group consists of members of the Hedgehog (Hh)-related-family, which are down-regulated in both data sets. The nematode Hh-related genes encode secreted proteins mostly expressed in the epidermis, which secretes the lipid- and collagen-rich cuticle, and in neurons and neuronal support cells required for the response to the environment [44-46]. Consistent with a possible role of these proteins in cuticle formation, inactivation of some Hh-related genes causes cuticle abnormalities and molting defects [45,46]. The co-regulation of Hh-related genes and structural components of the cuticle suggest that these genes could regulate the production of the dramatically different cuticles of dauer and reproductive L3 [47] through coordinated regulation by HPL-2. The third class of dauer and *hpl-2*-sensitive genes comprises a number of transporters whose expression is reduced upon dauer entry. These include members of the ABC transporter superfamily (*pgp-5*, 7 and *12*), and members of the MSF1 Major facilitator superfamily (F09A5.1, F14D7.6 and F53H8.3). Finally, the fourth and last class consists of cytochromes, enzymes involved in protection from toxins, hormone biosynthesis and other functions. Of the 81 members of the P450 family in *C. elegans*, 8 are present in the *hpl-2 *microarray. Three of these (*cyp-33*C9, *cyp-14*A5, and *cyp-37*B1) are down-regulated in *hpl-2 *mutants and in dauer, while two (*cyp-25*A2 and *cyp13*A5) are up-regulated in *hpl-2 *mutants and upon dauer entry [39,48]. Genes from each of these classes are also induced upon dauer exit [41], suggesting an important function in the regulation of both dauer entry and exit programs. HPL-2 binding and H3K9 methylation could be detected on a number of these dauer-regulated genes, including *cdh-10*, *cdh-12*, Y55F3AM.14, and *srr-2 *[49] (Figure 2), suggesting that they are likely to be direct targets of HPL-2.

### HPL-2 influences dauer entry and exit

The above results prompted us to test whether *hpl-2 *mutants are associated with dauer phenotypes. High temperature (27°C), scarce food and a secreted dauer pheromone influence dauer formation. While higher temperatures alone failed to induce a dauer phenotype in *hpl-2 *animals, when plates with identical bacterial lawns were seeded with the same number of worms at 25°C, we observed that starved plates containing *hpl-2 *mutant worms reproducibly had many more dauers than wild-type plates (data not shown). While this is not a quantitative assay, the observation nonetheless suggested that *hpl-2*-deficient animals enter dauer more easily under starved and crowded conditions at 25°C. We therefore tested whether *hpl-2 *mutants are more sensitive to dauer pheromone, an indicator of population density produced by the worms, which induces dauer formation at high concentrations.

At pheromone concentrations that trigger dauer formation in 23% of wild-type animals after 48 hours at 25°C, 97% of *hpl-2 *mutant animals entered dauer (Figure 3c). Therefore, *hpl-2 *mutants are associated with a hypersensitivity to dauer pheromone, which becomes evident under strong dauer-inducing conditions. When shifted to 20°C following exposure to dauer pheromone, a large majority of wild-type worms exit the dauer stage after 2 to 3 days. In contrast, only 15 to 20% of *hpl-2 *mutant worms exit dauer under the same conditions. We conclude that *hpl-2 *mutants exhibit both dauer entry and exit defects, namely hypersensitivity for dauer entry and a failure to exit efficiently.

The second *C. elegans *HP1 homologue, *hpl-1*, acts redundantly with *hpl-2 *in postembryonic development [24]. However, *hpl-1 *does not appear to act either alone or redundantly with *hpl-2 *in dauer development, since dauer formation was not impaired in either *hpl-1(tm1624) *single or *hpl-1(tm1624);hpl-2(tm1489) *double mutants at any of the temperatures tested.

### HPL-2 synergizes with the DAF-2/IIS pathway for dauer entry and exit

We next tested whether *hpl-2 *interacts genetically with either of the well-characterized dauer pathways: DAF-2/insulin-like signaling (IIS) and DAF-7/TGF-β. Mutations in either of these pathways have been isolated as dauer constitutive (daf-c), forming dauers even under non-dauer-inducing conditions [50]. We first constructed double mutants with the *daf-2(e1370) *allele, a well-established sensitized background for assaying a weak dauer phenotype [51]. While *daf-2 *single mutants do not form dauer at 20°C, 10 to 20% of double *hpl-2;daf-2 *mutants entered dauer at this temperature (Figure 3d, 20°C). This difference between single and double mutants was not detected at 22°C, a temperature at which single *daf-2(e1370) *mutants all enter dauer for approximately 3 days, then recover and resume development to become reproductive adults (data not shown). However, 3 days after egg-laying, when only 30% of the *daf-2 *single mutants remained as dauers, 98% of *hpl-2;daf-2 *double mutants were still in the dauer stage (Figure 3d, 22°C). Under these conditions, the block persisted for more than two weeks. At a slightly higher temperature, 25°C, 100% of the population remained in the dauer stage in both genetic backgrounds after 3 days (Figure 3d, 25°C). We conclude, therefore, that *hpl-2 *significantly enhances the dauer phenotype of *daf-2 *mutants.

Like *daf-2(e1370) *mutants, *daf-7(e1372) *mutants show a temperature-sensitive dauer constitutive phenotype [52]. At the semi-permissive temperatures of 15°C and 20°C, *hpl-2;daf-7 *double mutant animals show a greatly enhanced dauer arrest phenotype compared to single *daf-7 *mutants (Figure 3e). Our results thus suggest that HPL-2 acts in parallel or downstream to the IIS and TGF-β pathways to influence dauer through the regulation of genes important for both dauer entry and dauer exit processes. Given that this is a stress-induced differentiation event, our results implicate HPL-2 in communication between environment and a chromatin-controlled genetic response to stress.

### HPL-2 acts in the nervous system and intestine to influence dauer development

The decision to enter dauer is triggered by chemosensory stimuli. Two major head sensory organs, called amphids, are critical to dauer formation and both DAF-28/insulin and DAF-7/TGF-β are expressed in a subset of sensory neurons to impact dauer development [53-55]. These hormones in turn regulate the activity of widely expressed downstream receptors and transcription factors, which regulate dauer remodeling in other tissues. To establish in which tissue HPL-2 acts to influence dauer development, we carried out tissue-specific rescue experiments on *hpl-2;daf-2(e1370) *animals raised at 22°C (Figure 3f). While at this temperature all *daf-2 *animals reenter the reproductive cycle after 7 days, less than 1% of the double *hpl-2;daf-2 *mutants ever exit the dauer stage. Expression of *hpl-2 *under the control of either the pan neuronal *rab-3 *promoter or the intestinal *ges-1 *promoter allowed more than 30% of *hpl-2;daf-2(e1370) *mutant animals to exit the dauer stage. By contrast, expression of *hpl-2 *under the control of the *lin-31 *promoter, which is expressed in vulval precursor cells, failed to promote rescue. Furthermore, we confirmed that GFP expression of the *rab-3p::GFP::hpl-2 *rescuing transgene was limited to neuronal cells in *hpl-2;daf-2 *mutants (Additional file 7). These results suggest that the rescue observed with the *rab-3 *and *ges-1 *promoters is unlikely to be due to ectopic expression in additional cell types, but reflects the tissue-specific expression of these transgenes. The fact that rescue is partial could stem from insufficient induction of these promoters during dauer and/or from additional expression requirements in other tissues. Neuronal and intestinal expression together did not result in better rescue, however, suggesting that in these tissues HPL-2 does not have additive functions, but acts in the same process.

### *hpl-2 *modulates lifespan through the IIS pathway

**Figure 4:**
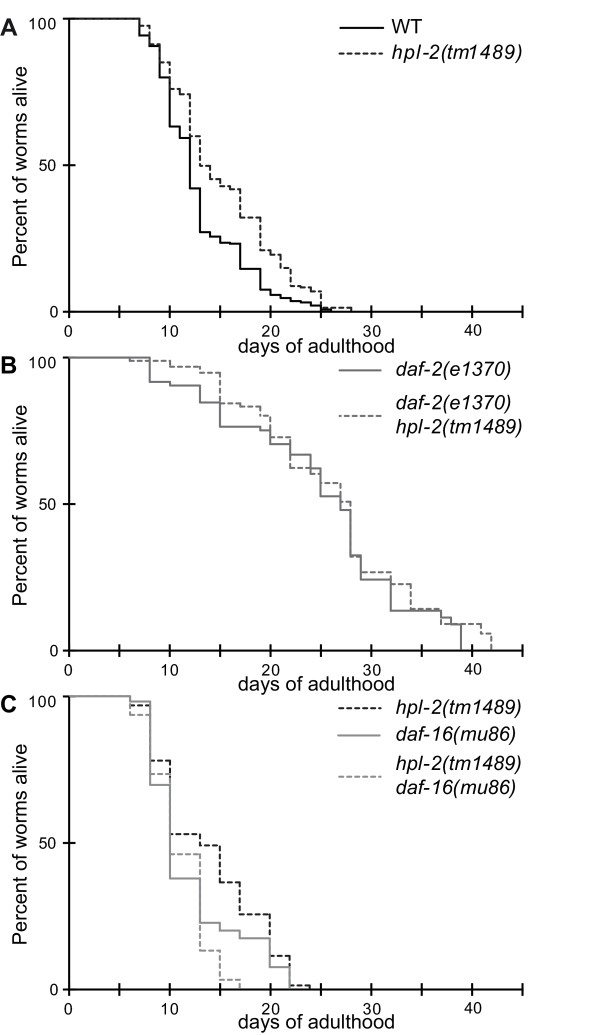
**HPL-2 modulates lifespan by acting through the *daf-2*/IIS pathway**. **(a) **Survival curve of wild-type and *hpl-2(tm1489) *animals. *hpl-2(tm1489) *mutation increases average lifespan by 17% (average lifespan: *hpl-2(tm1489)*, 15.1 ± 0.31 days (*n *= 287); wild type (WT), 12.9 ± 0.25 days (*n *= 280); log rank test *P *< 10^-3^). **(b) **Survival curve of *daf-2(e1370);hpl-2(tm1489) *double mutants compared to *daf-2(e1370) *single mutants. *hpl-2(tm1489) *did not further increase *daf*-*2(e1370) *mutant lifespan (average lifespan *daf-2(e1370)*, 26.2 ± 0.85 days (*n *= 95); *daf-2(e1370);hpl-2(tm1489)*, 24.8 ± 0.97 days (*n *= 84); log rank test *P *= 0.271.). **(c) **Survival curve of *daf-16(mu86);hpl-2(tm1489) *compared to *daf-16(mu86). daf-16(mu86) *mutation suppressed *hpl-2(tm1489) *mutant lifespan phenotype (average lifespan: *daf-16(mu86)*, 11.9 ± 0.44 days (*n *= 112); *daf-16(mu86);hpl-2(tm1489)*, 11.03 ± 0.27 days (*n *= 109); log rank test *P *= 0.05).

Our results suggest that *hpl-2 *synergizes with the IIS pathway to control dauer development. Given that the IIS pathway is also a well-established regulator of lifespan in *C. elegans*, *Drosophila *and mice [56], we performed lifespan analysis of *hpl-2 *null mutants at the permissive temperature (20°C). We observed a reproducible increase in the mean lifespan of *hpl-2 *animals by 17% on average (average lifespan: *hpl-2(tm1489)*, 15.1 ± 0.31 days (*n *= 287); wild-type, 12.9 ± 0.25 days; log rank test *P *< 10^-3^; Figure 4a). To confirm that the increased lifespan is due to inactivation of *hpl-2 *and not to a background mutation, we repeated lifespan assays of animals in which *hpl-2 *was inactivated by RNAi feeding. *hpl-2(RNAi) *only partially inactivates *hpl-2 *activity and compared to *hpl-2 *loss of function results in weaker phenotypes only apparent at higher temperatures [22,23]. However, similarly to the *hpl-2 *mutant, longevity of *hpl-2(RNAi) *animals raised at 20°C and shifted to 25°C at the L4 stage was extended by 18% on average compared to control animals (Additional file 8), confirming that HPL-2 is required for normal longevity. Lifespan extension in the absence of HPL-2 is unlikely to be related to a germline proliferation defect, as *hpl-2 *mutants have a wild-type brood size and germline cell number at 20°C [22,23] (Additional file 9).

To assess whether the lifespan increase observed in *hpl-2 *mutants depends on the IIS pathway, we analyzed the lifespan of *hpl-2;daf-2 *double mutants. The two-fold increase in lifespan of *daf-2(e1370) *mutants was unchanged in *hpl-2;daf-2 *animals (average lifespan: *daf-2(e1370)*, 26.2 ± 0.85 days (*n *= 95); *daf-2(e1370);hpl-2(tm1489)*, 24.8 ± 0.97 days (*n *= 84); log rank test *P *= 0.271; Figure 4b). Therefore, the *hpl-2 *lifespan phenotype may rely on IIS signaling. The transcription factor DAF-16/FOXO acts downstream of *daf-2*/IIR and is essential for lifespan extension by *daf-2*. We therefore tested whether the *hpl-2 *mutant lifespan phenotype also requires DAF-16/FOXO. *hpl-2(tm1489)*;*daf-16(mu86) *mutants had a lifespan indistinguishable from that of *daf-16(mu86) *alone, which is 16% shorter than wild-type worms (average lifespan: *daf-16(mu86)*, 11.9 ± 0.44 days (*n *= 112); *daf-16(mu86);hpl-2(tm1489)*, 11.03 ± 0.27 days (*n *= 109); log rank test *P *= 0.05; Figure 4c). Furthermore, the *hpl-2 *effect on longevity is not due to altered expression of genes encoding proteins involved in the IIS pathway, as the levels of *daf-16 *and other major components of the pathway, including *daf-2*, *age-1*, *akt-1*, *akt-2 *and *daf-18*, were not altered in *hpl-2 *mutants (Additional file 1). Taken together, our results suggest that *hpl-2 *requires the activity of *daf-16 *to modulate longevity in *C. elegans *and that *hpl-2 *may be a novel co-regulator of *daf-16*-controlled genes.

To better define this relationship, we looked for overlap with the 514 genes that microarray analysis by Murphy *et al*. [57] found to be modulated by DAF-16; 46 of the genes that we found to be misregulated in the absence of *hpl-2 *activity overlap with genes regulated by DAF-16. Of these, 16 are genes repressed in *hpl-2 *and in long-lived *daf-2 *pathway mutants, and activated in *daf-2;daf-16 *mutants, reflecting DAF-16 dependence (Additional file 2e; *P *= 0.003). We also compared our dataset to a recent transcriptional profile to identify genes whose expression changes over the *C. elegans *lifespan [58]. Out of 1,217 ageing-associated genes, 66 were down-regulated in the absence of *hpl-2*, representing a significant enrichment (Figure S2f; *P *= 5 × 10^-7^). Taken together, these analyses suggest that the effect of *hpl-2 *on longevity may be mediated, at least in part, through the regulation of longevity-associated genes, some of which may be DAF-16-dependent.

### *hpl-2 *mutants do not show altered *daf-16 *localization or increased stress resistance

Similar to mammalian FOXO transcription factors, translocation of DAF-16 from the cytoplasm to the nucleus is required for activation, and has been observed in animals with reduced DAF-2/IIS signaling and in response to stresses such as heat shock or starvation [59]. Using a previously described DAF-16::GFP reporter [60], we observed no change in DAF-16 localization in animals lacking HPL-2 (data not shown and Additional file 10). Therefore, HPL-2 does not appear to impinge on longevity by altering DAF-16 localization.

Many long-lived mutants also exhibit increased resistance to environmental stress. However, *hpl-2 *mutants are not significantly more resistant to oxidative stress, as measured on paraquat (Additional file 11), and are intrinsically thermosensitive. Therefore, the increased longevity of *hpl-2 *mutants appears to be uncoupled from increased resistance to environmental stressors, as already observed in other contexts [55,61-64]. Consistently, we did not observe increased expression of genes commonly associated with increased stress resistance - for example, the *mtl-1 *metallothionein gene and the mitochondrial *sod-3 *superoxide dismutase gene - while expression of the heat shock proteins *hsp-16 *and *hsp-12 *actually decreased [65-67] (Additional file 1).

### *hpl-2 *regulates fat metabolism

**Figure 5:**
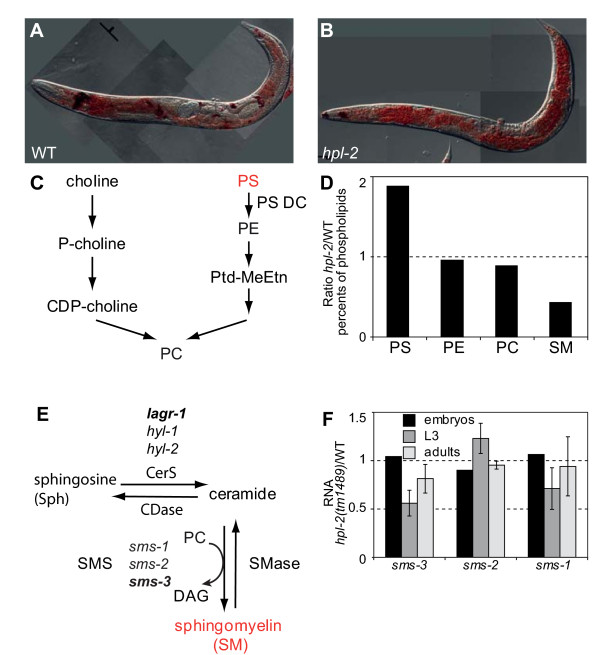
**HPL-2 is a regulator of phospholipid metabolism**. **(a, b) **Oil Red O representative images of wild type (WT) (a) and *hpl-2(tm1489) *(b) animals. Worms are F1 progeny of L4 hermaphrodites shifted to 25°C. Images were taken with a CoolSnap Color Camera with 700 ms exposure time. Scale bar = 50 μm. **(c) **Simplified overview of phosphatidyl choline (PC) metabolism in *C. elegans*. P-choline, phosphorylcholine; CDP-choline, cytidyldiphosphate choline; PC, phosphatidylcholine; PS, phosphatidylserine; PS DC, phosphatidylserine decarboxylase; PE, phosphatidylethanolamine; Ptd-MeEtn, phosphatidylmonomethylethylamine. Intermediates whose levels are altered in *hpl-2 *mutant worms compared to wild type are shown in red. **(d) **Phospholipid content analysis in wild-type and *hpl-2 *mutant worms. Bars represent the amount of each lipid species found as a percentage of the total phospholipid content. PS, phosphatidylserine; PE, phosphatidylethanolamine; PC, phosphatidylcholine; SM, phingomyelin. Data are the average of two independent cultures. **(e) **Metabolism of sphingomyelin and ceramide. *De novo *sphingomyelin synthesis is mediated by sphingomyelin synthase (SMS), which transfers the phosphorylcholine moiety from phosphatidylcholine (PC) onto ceramide, forming sphingomyelin and diacylglycerol (DAG). In *C. elegans *there are three SMS genes, *sms-1*, -*2*, and -*3*. Ceramide can be generated either by the action of sphingomyelinase (SMase) or by *de novo *synthesis through the enzyme ceramide synthase (CerS, *lagr-1*, *hyl-1 *and *hyl-2 *in *C. elegans*). **(f) ***hpl-2 *regulates *sms-3 *mRNA levels. *hpl-2*/N2 ratios for sphingomyelin synthase *sms *genes at different developmental stages. Experiments were performed on three independent synchronized L3 cultures and on two adult and embryonic cultures. Reference genes are *pmp-3 *and *cdc-42*. Normalization was performed using the mean of both reference genes. Data are the mean of independent biological replicas.

Adipose tissue has been implicated in the regulation of longevity in a number of species [68-70]. In *C. elegans*, lipid hydrolysis in fat storage tissue was recently shown to prolong life span, connecting the metabolic functions of adipose tissue to lifespan control [71]. Genes associated with fatty acid metabolism are over-represented in the set of *hpl-2 *down-regulated genes (Table 1), prompting us to test whether *hpl-2 *mutants may have defects in lipid metabolism that could contribute to the longevity phenotype. Down-regulated genes include the *fat-7 *Δ9 fatty acid desaturase, *acs-2*, an acyl-CoA synthetase, and *ech-1*, an enoyl-CoA hydratase. *hpl-2 *mutants raised at the non-permissive temperature are transparent, resembling starved animals (data not shown). This 'clear' phenotype has been associated with decreased fat storage in the intestinal compartment [72]. However, using the neutral lipid dye Oil red O [73], no reproducible difference in staining intensity was observed between wild-type and *hpl-2 *mutant animals at either permissive or non-permissive temperatures (Figure 5a, b and data not shown), ruling out any gross alteration in lipid content.

Quantitative lipid analysis following solid phase extraction [74] revealed more subtle, highly reproducible differences in lipid composition in *hpl-2 *mutant animals at the permissive temperature. Consistent with the Oil red O staining results, no difference in the total content and composition of neutral lipids was observed and levels of triacylglycerides and fatty acids were unchanged in *hpl-2 *animals compared to wild type (Additional file 12 and data not shown). In contrast, phospholipid analysis showed significant changes in phospholipid content (Figure 5d). Higher amounts of phosphatidylserine (PS) were observed in *hpl-2 *mutant animals, whereas no increase in phosphatidylethanolamine (PE), the decarboxylation product of PS, was observed. These results may therefore reflect changed expression or function of PS decarboxylase leading to lower synthesis of PE. Phosphatidylcholine (PC) is the predominant membrane lipid in eukaryotes and also functions in signal transduction pathways. Although amounts of PC were slightly reduced in *hpl-2 *mutant worms, the fact that we do not observe any obvious defects in *hpl-2 *mutants at the permissive temperature suggests either that the observed decrease in PC is not large enough to impinge on PC mediated processes, or that compensatory mechanisms exist.

Of the different classes of phospholipids analyzed, the most dramatic change was observed for sphingomyelin (SM), whose content was reduced by more than 60% in *hpl-2 *mutants. SM is a vital component of cellular membranes and plays an important role in signal transduction and membrane trafficking [75]. Synthesis of SM involves a transfer of phosphorylcholine from PC to ceramide by sphingomyelin-synthases (SMSs; Figure 5E). Expression of *lagr-1*, encoding a ceramide synthase (CerS), is decreased in *hpl-2 *mutant animals. This could potentially result in lower levels of ceramide, thereby influencing SM levels. However, the total content of ceramides (hydroxylated and nonhydroxylated) was not affected in the *hpl-2 *mutant context (Additional file 12). Therefore, the decrease in SM levels is more likely to be due to the down-regulation of *de novo *SM synthesis rather than enhanced degradation. *C. elegans *contains three SMS homologues, one of which (*sms-3*) is down-regulated in *hpl-2 *mutants. qRT-PCR analysis confirmed that *sms-3 *RNA levels are decreased approximately two-fold with respect to wild type in L3 animals, with a smaller effect in young adults (Figure 5f). Similarly, *sms-1 *levels also appeared to be slightly reduced in L3, but no significant change in *sms-2 *RNA levels was observed, nor were changes in *sms-3 *or *sms-1 *levels observed at the embryonic stage. As shown in Figure 2, ChIP experiments revealed a small but reproducible enrichment of HPL-2 binding within the *sms-3 *coding regions in L3 larvae. Therefore, it is likely that HPL-2 directly influences SM levels through the regulation of *sms-3 *transcription.

## Discussion

Because *C. elegans *chromosomes are holocentric and lack centromeric heterochromatin, HP1 proteins are dispensable for chromosome segregation and mitotic viability in this organism [23,25]. We have exploited this fact to examine in detail the role of one of the *C. elegans *HP1 homologues, HPL-2, in regulating developmental pathways of gene expression, a second physiological function ascribed to heterochromatin. By comparing the effects of *hpl-2 *deletion at different stages of development, we have shed light on a novel role for this HP1 family member in dauer diapause, longevity and lipid metabolism. These three processes have in common an extremely tight response to environmental factors. Thus, our data link a response to environmental factors to the epigenetic regulator HPL-2. At least some of the genes identified in this study are shown by CHIP-on chip to be bound by HPL-2, and are therefore likely to represent direct targets.

We show that *hpl-2 *plays a role in the choice between dauer and non-dauer development, a striking example of phenotypic plasticity in which environmental conditions determine developmental fate. Many of the genes down-regulated in *hpl-2 *mutant larvae at the L3 stage show high expression in wild-type L3 and dramatic reduction in dauer. These include the cadherins *cdh-10 *and *cdh-12*, the metalloprotease *nas-27 *and the collagen *col-104 *[49]. Conversely, genes up-regulated in *hpl-2 *mutants often show low expression in wild-type L3 and strong expression in dauer. Down-regulation of metalloproteases, collagen genes, and Hh-related peptides in *hpl-2 *mutants most likely reflects synthesis and remodeling of the cuticle, an important feature of the dauer program. Their down-regulation in *hpl-2 *mutants may facilitate entry into dauer and impair dauer exit, which are the two phenotypes we observe in animals lacking HPL-2.

Recently, post-dauer animals have been shown to have markedly altered genome-wide histone tail modifications, as well as modified gene expression profiles [76]. Furthermore, mutations of genes implicated in chromatin function, including *hpl-2*, were found to abolish or alter changes in gene expression between control and post-dauer animals. Remodeling of chromatin architecture may therefore play an essential role in the establishment or maintenance of the post-dauer expression changes. The inability of *hpl-2 *worms to exit dauer suggests that *hpl-2 *also functions during the dauer stage to activate the transcriptional program required to exit this developmental stage and re-enter the reproductive life cycle. Consistently, we also found a significant overlap between genes down-regulated in the absence of HPL-2 and genes whose expression increases upon dauer exit.

HPL-2 functions in dauer through the DAF-2/IIS and the TGF-β pathways. The decision to enter or exit dauer is mediated by these neuroendocrine signals acting through sensory neurons [40,77]. These in turn regulate the developmental and metabolic shifts in remodeled tissues. Although HPL-2 is ubiquitously expressed, we show using cell type-specific rescue that HPL-2 is sufficient in either the nervous or the intestinal system to induce partial rescue of the dauer exit phenotype. In the nervous system, it may function to regulate the activity of widely expressed downstream receptors and transcription factors, while in intestinal cells it may more directly mediate the dramatic reshaping of this tissue, a process that can be separated from the remodeling of other tissues [78]. Supporting a cell autonomous function for HPL-2 in neuronal cells, we find that neuronally expressed genes are significantly overrepresented in the *hpl-2 *microarray set (Additional file 3; FET *P *< 10^-9 ^for up-regulated genes, *P *< 3.10^-3 ^for down-regulated genes). In both tissues, HPL-2 appears to be important for environmentally induced changes in tissue-modifying gene expression.

We also show that mutation of *hpl-2 *leads to a 17% increase in longevity. RNAi-based screens have identified a number of additional chromatin regulators as being important components of longevity, including the *set-9 *and *set-15 *putative H3K9 methyltransferases [79]. More recently, members of an H3K4 histone methylation complex were also found to regulate lifespan [80], suggesting that epigenetic mechanisms are likely to play an important role in the ageing process. In *Drosophila*, changes in *HP1 *copy number were not found to have any effect on longevity [81]. However, whole genome profiling has shown a dramatic redistribution of HP1 and H3K9 methylation in aging flies [82]. Similarly, aging human cells show decreased H3K9me3 and HP1γ staining [83]. Although the specific functional consequences of these age-related changes have not been directly determined, changes in gene expression are likely to be involved.

Here we demonstrate that lifespan extension by mutation of *hpl-2 *depends on both DAF-2 and DAF-16/FOXO. DAF-16 is a key regulator of longevity, metabolism and dauer diapause, and several microarray experiments have been carried out to identify potential DAF-16 targets. The requirement for *daf-16 *activity for the *hpl-2 *longevity phenotype could reflect regulation of some of the same target genes. Although all of the published *daf-16 *microarray studies have been carried out on adult animals [57,84,85], while our analysis was carried out on early L3 larva, we were nonetheless able to observe a limited overlap between potential DAF-16 targets identified by Murphy *et al*. [57] and *hpl-2 *regulated genes. Some of these same genes are also regulated upon dauer entry, suggesting that HPL-2 may act in dauer and ageing at least in part through a common mechanism that is dependent on DAF-16.

The longevity of *hpl-2 *mutants may also be related to the function of HPL-2 in repressing germ-cell fate in the soma. A significant number of genes up-regulated in L3 larvae in the absence of *hpl-2 *are germline-expressed genes, and like long-lived *daf-2*/IIS mutants [27], *hpl-2 *mutants show features of soma-to-germline transformation [28,33]. Soma-to-germline transformation is common to a number of transcriptional repressors, including the worm retinoblastoma homolog LIN-35, E2F components, and members of the worm NuRD complex, suggesting that HPL-2 may play a direct role in maintaining germline genes in a repressive chromatin state [28,33].

The altered levels of lipid metabolic enzyme transcripts in *hpl-2 *mutants may represent an additional mechanism relevant to the observed lifespan increase. HPL-2 regulates the expression of genes involved in fatty acid and phospholipid metabolism. In mammals, glucose and lipid homeostasis in almost all major metabolic tissues is regulated in response to nutrient availability by the activity of the histone deacetylases SIRT1 [86]. Overexpression of the SIRT1 homolog Sir2 extends lifespan in *Saccharomyces cerevisiae*, *C. elegans *and *Drosophila *[87-89], possibly by altering lipid homeostasis through the conserved lipid/cholesterol regulator SREBP [90,91]. Our data suggest that HPL-2 may regulate phospholipid metabolism through regulation of two key enzymes: the sphingomyelin synthase gene *sms-3 *and the ceramide synthase *lagr-1*. Expression of *sms-3 *may be directly up-regulated by HPL-2, as we are able to detect HPL-2 binding to the *sms-3 *coding region. This association is consistent with growing evidence suggesting that HP1 proteins can also play positive roles in transcription through binding within gene bodies [5,11,13,92].

Reduced expression of *sms-3 *and *lagr1 *in the absence of HPL-2 is associated with a 60% reduction in SM levels. Sphingolipids, including SM and its precursor ceramide, are cellular membrane components found in all eukaryotes and implicated in numerous functions, including the cellular response to environmental stresses and ageing [75,93-95]. In yeast, the ceramide synthase component *LAG1 *was one of the first longevity genes characterized [96], and subtle changes in ceramide/sphingolipid metabolism are important in determining yeast longevity [97]. *lagr-1 *encodes one of three *C. elegans *orthologues of yeast LAG1. *lagr*-*1(RNAi) *results in reduced fat content and suppresses alpha-synuclein inclusions in living and aging animals, a phenotype associated with increased longevity [98]. Although neither *lagr-1 *nor *sms-3 *loss of function alone is associated with increased longevity [99] (FP and YX, data not shown), decreasing expression of *hyl-1*, another worm homolog of *LAG1*, increases longevity [99,100], and this effect is enhanced in *hyl-1;lagr-1 *double mutants (N Faergeman, personal communication). Therefore, altered phospholipid metabolism in the absence of *hpl-2 *activity might contribute directly to the longevity phenotype associated with *hpl-2 *mutant animals.

Taken together, our results highlight the importance of epigenetic regulation in the maintenance of homeostasis. As an epigenetic regulator, *hpl-2*/HP1 could both stabilize developmental decisions by buffering environmental variation, as well as guide the organism through remodeling events such as dauer that require plasticity of cell fate regulation. The signaling pathways that link HPL-2 to environmental cues are under investigation.

## Conclusions

*C. elegans *provides an optimal system to explore the roles of HP1 beyond centromeric heterochromatin because worm chromosomes are holocentric and lack the alpha satellite repeats that nucleate the binding of HP1 to centromeres in other species. We have compared expression profiles of wild-type worms and worms lacking HLP-2, one of this organism's two HP1 homologues, at two developmental stages. We find that HPL-2 regulates the expression of germline genes, extracellular matrix components and genes involved in lipid metabolism. We show that in the soma, HPL-2 is involved in the dauer developmental decision, which is tightly linked to environmental conditions. We further uncover a novel function for HP1-regulated gene expression in longevity and lipid homeostasis. These pathways share the characteristic of responding to environmental cues. We suggest that HPL-2 influences the expression of genes that regulate developmental processes in response to environmental conditions.

## Materials and methods

### Strains and genetics

Strains were maintained according to the standard protocol [101]. The following mutant alleles and transgenic strains were used: LGI, *daf-16(mu86)*; LGIII, *hpl-2(tm1489)*, *daf-2(e1370)*, *daf-7(e1372)*; LGIV, *zIs356 (daf-16::gfp)*. The Bristol strain N2 was used as wild type. To construct the *hpl-2(tm1489); zIs356 (daf-16::gfp*) strain, *zIs356 (daf-16::gfp) *hermaphrodites were mated with *hpl-2 (tm1489) *males. F2 worms were screened for the presence of GFP, and the presence of the *hpl-2 *deletion allele was confirmed by PCR analysis as previously described [23].

### Isolation of RNA for microarray analysis

Wild-type and *hpl-2(tm1489) *gravid adult animals were synchronized by bleaching. Half of the culture was collected immediately for RNA extraction from the embryos. The other half was left to hatch overnight in M9 without food. Synchronized L1 larvae were then distributed onto peptone plates, grown at 20°C and staged until they reached the L3 larval stage (between 28 and 29 hours). The developmental stage was confirmed by microscopic observation, using both vulval development and somatic gonad development as criteria.

Triple biological replicates were flash frozen in liquid N2 and RNA was extracted using the standard Trizol method followed by RNeasy column purification (Qiagen, France, #74104). RNA was labeled and hybridized to Affymetrix tiling arrays. Washing and scanning were performed following the manufacturer's instructions. Oligos from the tiling array were mapped to chromosome coordinates of the exons from Wormbase WS180. Any oligo that mapped to a gene on both the Watson and Crick strands was excluded. The remaining oligos were then grouped together (perfect match and mismatch) into probe sets and written out into an Affymetrix CDF file. The CDF file was converted into an R-package and loaded into R. The expression values were calculated using the justRMA function from Bioconductor. This used a Benjamini and Hochberg false discovery rate correction. The R code is available upon request.

### Quantitative PCR on reverse transcription products

cDNAs were synthesized with a mix of random hexamers and oligo dT primers using iScript cDNA synthesis kit and 500 ng of RNA (BIO-RAD, France). qPCR reactions on reverse transcription products diluted ten times were performed using FastStart Universal SYBR Green Master (Roche, France) and the StepOne Plus Detection System (Applied Biosystems, France). The qPCR conditions were 95°C for 4 minutes, followed by 40 cycles of 12 s at 95°C and 30 s at 60°C. Melting curve analysis was performed for each primer set at the end to ensure the specificity of the amplified product. Standard curves were generated for each primer set to calculate the efficiency of each set. Only primer sets with an efficiency between 1.9 and 2.1 were used for qPCR. Primer sets for the *pmp-3 *and *cdc-42 *transcripts were used as internal controls, and the RNA level of each gene of interest was normalized to the level of the mean of both these genes for comparison. qPCR experiments were repeated at least three times using independent RNA/cDNA preparations. Data were pooled and analyzed using the StepOne Plus Software (Applied Biosystems). Primer sequences are available upon request.

### Comparisons of different microarray datasets

The *P*-values for overlaps between lists were generated by comparison to results of random simulation and by calculation based on the hypergeometric distribution as in [102], using R.

### Construction of transgenic lines

For intestinal expression, the *hpl-2 *(K01G5.2a) full-length cDNA was cloned into the XbaI-SacII sites of pJM16 under the control of the *ges-1 *promoter [103]. For pan-neuronal expression, the cDNA was cloned into the NotI-SmaI sites of pHU004, under the control of the *rab-3 *promoter in fusion with GFP (gift from JL Bessereau). Both intestinal-specific and neuronal-specific constructs, named pCB15 and pSS05, respectively, were injected into N2 hermaphrodites at a concentration of 5 mg/ml using pRF4 as a co-transformation marker at a concentration of 200 mg/ml. For each construct, two to three independent transgenic lines were then crossed with *hpl-2(tm1489);daf-2(e1370) *males. F2 rollers were allowed to lay eggs at 15°C, then shifted to 25°C. The F3 were selected for both 100% dauer in the progeny and the presence of the *hpl-2 *deletion allele.

### Lifespan assay

Lifespan tests were performed at 20°C as described in [104]. For each genotype, lifespan experiments were repeated two to four times. Survival analysis was carried out using the Kaplan Meier method and the significance of differences between survival curves calculated using the log rank test. The statistical software used was XLSTAT 2007 and all *P*-values < 0.05 were considered significant.

### Chromatin immunoprecipitation

The ChIP protocol was based on [105], with a number of modifications. L3 staged wild-type or transgenic animals carrying an integrated rescuing *hpl-2*::GFP construct [23] were used as starting material. Worm powder was incubated in phosphate-buffered saline containing 1.5 mM EGS for 20 minutes at room temperature, followed by the addition of formaldehyde (1% final) for 10 minutes at room temperature. After quenching with glycine (125 mM final), the worm pellet was washed in phosphate-buffered saline, resuspended in lysis buffer (50 mM HEPES/KOH pH 7.6, 1 mM EDTA, 0.5% sarkosyl, 150 mM KCl, protease inhibitors cocktail (Roche Biochemicals) and 1 mM PMSF) and sonicated in a Bioruptor water bath (Diagenode, Belgium). The homogenate was centrifuged at 12,000 rpm for 15 minutes at 4°C and the supernatant containing the sheared chromatin (chromatin extract) precleared twice for 30 minutes at 4°C with 80 μl of a 1:1 slurry of protein A agarose beads (Millipore #16157, France). The cleared extract was diluted with 2 volumes of FA buffer (50 mM HEPES/KOH pH 7.5, 1 mM EDTA, 1% Triton X-100, 0.1% sodium deoxycholate, 150 mM KCl, protease inhibitors cocktail and 1 mM PMSF). Following incubation with antibodies (anti-HPL-2 or anti-GFP), 45 μl of a 1:1 slurry of protein A agarose beads was added to each tube for 1 hour. Beads were washed twice with FA buffer, once with FA-500 (50 mM HEPES/KOH pH 7.5, 1 mM EDTA, 1% Triton X-100, 0.1% sodium deoxycholate, 500 mM NaCl), once with FA-1M (50 mM HEPES/KOH pH 7.5, 1 mM EDTA, 1% Triton X-100, 0.1% sodium deoxycholate, 1 M NaCl), once with TEL (0.25 M LiCl, 1% NP-40, 1% sodium deoxycholate, 1 mM EDTA, 10 mM Tris-HCl, pH 8.0), and twice with TE (10 mM Tris/HCl, 1 mM EDTA). Elution was performed by adding two times 100 μl of elution buffer (10 mM Tris/HCl, 1 mM EDTA, 250 mM NaCl, 1% SDS) and incubating at 65°C for 10 minutes. Supernatants were pooled, incubated for 30 minutes with 1 μl of RNase A (10 mg/ml) and then incubated overnight at 65°C with 1 μl of proteinase K (15 to 20 mg/ml). DNA was recovered using a Qiaquick pure purification kit in 40 μl of PCR grade water for qPCR analysis. ChIP results were analyzed by qPCR using primers specific to the *sms-3 *locus. A primer corresponding to an intergenic region on chromosome IV was used as control. The anti-GFP antibody used was from Abcam, UK (#ab290).

### Lipid extraction and analysis

The solid phase extraction procedure was performed as previously described [74]. Briefly, lipids were extracted using the Folch procedure [106] and loaded onto a 100 mg aminopropyl LC-NH2 cartridge pre-equilibrated with 3 ml of hexane, and placed onto a vacuum manifold apparatus (12 port Visiprep, Supelco, Sigma-Aldrich, France). Neutral lipids (sterols, sterol esters, methylated fatty acids, tri- and diacylglicerides; fraction 1) were eluted with 1.4 ml hexane:ethylacetate (85:15 v/v) at a solvent flow rate of 0.3 ml/minute obtained by applying negative pressure. Ceramides (fraction 2) were eluted with 1.6 ml of chloroforme:methanol (23:1 v/v). Free fatty acids (fraction 3) were eluted with 1.8 ml diisopropylether-acetic acid (98:5, v/v), and this fraction was pooled together with fraction 1. Glycosphingolipids (fraction 4) were eluted using 2.1 ml of acetone-methanol (9:1.35, v/v). Neutral and acidic phospholipids were eluted together in one fraction (fraction 5) using 2 ml of methanol. The fractions were dried under N_2 _and resolved by thin layer chromatography using the following solvent systems: for separation of neutral lipids and free fatty acids, hexane:diethylether:acetic acid (80:20:1 v/v/v); for ceramides, chloroform:methanol (50:5 v/v); for glycosphingolipids, chloroform:methanol:water (65:25:4 v/v/v); for resolution of phospholipids by two-dimensional thin layer chromatography, tetrahydrofurane:acetone:methanol:water (50:20:40:6 v/v/v/v) in the first direction and chloroform:acetone:methanol:aceticacid:water in the second direction (50:20:10:15:5 v/v/v/v/v). Fractions 1, 2, 3 and 4 were detected after spraying with cupric sulfate and charred at 180°C for 5 minutes. Phospholipids were detected by iodine and scraped into glass tubes. Lipidic phosphorous was mineralized by warming the samples in 0.25 ml mixture of sulfuric acid:perchloric acid (2:1 v/v) containing vanadium tetraoxide (1 g/l) for 20 s above the flame and quantified by 5 ml of reagent containing 0.63 g of ANSA mixture, 0.2 g ammonium heptamolybdate in 100 ml of water (ANSA contains 60 g of sodium metabisulfate, 2 g of sodium sulfate and 1 g of 1-amino 2-naphtol 4-sulfonic acid). Samples were mixed by vortexing, incubated at 100°C for 8 minutes and absorbances were read at 830 nm. Standard curves were made by the use of monopotassium phosphate.

### Data availability

Microarray data are publicly available through NCBI, Gene Expression Omnibus accession number [GSE33700].

## Abbreviations

ChIP: chromatin immunoprecipitation; FET: Fisher exact test; GFP: green fluorescent protein; GO: Gene Ontology; H3K9: histone H3 lysine 9; H3K9me3: tri-methylated lysine 9 of histone H3; Hh: Hedgehog; Hp: Heterochromatin protein; IIR: insulin/insulin-like growth factor-I-like receptor; PC: phosphatidylcholine; PE: phosphatidylethanolamine; PMSF: phenylmethanesulfonylfluoride; PS: phosphatidylserine; qPCR: quantitative PCR; qRT-PCR: quantitative reverse-transcriptase PCR; RNAi: RNA interference; SM: sphingomyelin; TGF: transforming growth factor.

## Competing interests

The authors declare that they have no competing interests.

## Authors' contributions

PM participated in the design of the study, analyzed the data, performed the statistical analysis, and helped to draft the manuscript. SS carried out genetic analysis. CB and BH carried out the molecular analysis. YX carried out the molecular analysis and the lipid analysis. LM and FS carried out the longevity assays. SB carried out the lipid analysis. SG helped draft the manuscript. FP conceived the study, participated in its design and coordination and drafted the manuscript. All authors read and approved the final manuscript for publication.

## Supplementary Material

Additional file 1 **Differentially expressed genes for embryos and L3 stage larvae in wild type compared to *hpl-2 *mutant animals**. Click here for file

Additional file 2 **Increased *lin-13*::GFP expression in the absence of HPL-2 activity**. Expression of a *lin-13*::GFP translational fusion [108] in wild-type and *hpl-2 *mutant animals. In *hpl-2 *mutant animals GFP expression was particularly strong in nuclei in the head region (arrows). Click here for file

Additional file 3 **HPL-2 regulation of specific gene classes. (a-f) **Venn diagrams showing overlap between: (a) genes up-regulated in L3 stage *hpl-2 *mutants and germline genes; (b) genes down-regulated in L3 stage *hpl-2 *mutants and genes induced in dauer exit; (c) genes down-regulated in L3 stage *hpl-2 *mutants and genes clustered in expression mountain 29 of the *C. elegans *three-dimensional topographical expression map; (d) genes regulated in L3 stage *hpl-2 *mutants and neuronally expressed genes; (e) genes down-regulated in L3 stage *hpl-2 *mutants and *daf-16 *target genes; (f) genes down-regulated in L3 stage *hpl-2 *mutants and aging regulated genes. Click here for file

Additional file 4 **Overlap between *hpl-2*-regulated genes and genes bound by HPL-2**. Venn diagram showing overlap between genes regulated in L3 stage *hpl-2 *mutants and genes bound by HPL-2 [29]. Click here for file

Additional file 5 **HPL-2 and H3K9me3 are enriched on repetitive transgenes. (a) **Histogram representing the percentage of immunoprecipitation, calculated as the ratio between signal from the antibodies and signal from pre-immune serum, from ChIP experiments along a repetitive transgene. Synchronized populations of L3 wild-type or *hpl-2 *mutant worms carrying the trangene were subjected to immunoprecipitation using anti-HPL-2, anti-H3K9me3, or pre-immune serum as control. **(b) **Schematic representation of the transgene used in these experiments [109] showing the position of the primers used for qPCR analysis. Similar results were obtained for three to four independent experiments. Click here for file

Additional file 6 **Repressive histone mark levels on promoters and genes misregulated in *hpl-2 *mutant worms**. For each gene or gene promoter, H3K9 mono-, di- and tri-methyl levels were calculated as the median level observed on the DNA stretch using data provided by the modENCODE Consortium [29]. The criterion for choosing control genes was similar expression levels. Data are presented as box-whisker representation where the bar indicates the median of all up- or down-regulated genes and the upper and lower edges of the box represent the 25th and 75th percentiles, respectively. Up-regulated genes show a significantly higher level of mono-, di- and tri-methylated H3K9, but no enrichment in H3K27 trimethylation, compared to expression-matched control genes. Down-regulated genes show a similar level of mono-, di- and tri-methylated H3K9 compared to expression-matched control genes. Click here for file

Additional file 7 ***rab-3p*::*hpl-2cDNA::GFP *transgene is expressed uniquely in neuronal cells**. GFP fluorescence is detected in the synaptic-rich regions of the nervous system, including the nerve ring, ventral nerve cord, and dorsal nerve cord (arrowheads). Click here for file

Additional file 8 **Increased lifespan of *hpl-2(RNAi) *animals**. Animals at the L4 stage were placed on RNAi or control bacteria at 20°C and allowed to lay eggs. When F1 progeny reached the L4 stage, they were transferred to 25°C for lifespan assays on plates containing 10 μM 5FU. Worms were then transferred to fresh RNAi plates every week. *hpl-2 *RNAi increased average lifespan by 18% (average lifespans: wild type fed control bacteria, 10.3 ± 0.42 (*n *= 60); wild type fed *hpl-2 *RNAi clone 1, 12.2 ± 0.32 days (*n *= 60; control comparison *P *= 10^-3^). Similar results were obtained in two independent tests. Click here for file

Additional file 9 ***hpl-2 *deletion does not affect the total number of germ cells produced**. Data are displayed as the mean (± standard deviation) number of germ cells per gonad arm (*n *= 4). Germ cell number was obtained by counting germ cell nuclei stained with DAPI in dissected gonads of young adults. Click here for file

Additional file 10 ***hpl-2 *does not influence DAF-16::GFP localization**. Adults carrying a *daf-16::gfp *transgene (*zIs356*) were placed on NGM plates at 37°C. After 45 minutes, worms were mounted onto a slide in M9 buffer. Nuclear translocation of DAF-16::GFP was visualized with a fluorescence microscope AxioImager Z1 (Zeiss) equipped with a CoolSnap HQ camera and driven by Metamorph software (Molecular Devices, France). Click here for file

Additional file 11 **The *hpl-2(tm1489) *deletion mutant does not affect resistance to oxidative stress**. Adult hermaphrodites were incubated in M9 buffer with 100 mM paraquat. After incubation at 20°C for the specified duration, survival was measured. Worms were scored as dead when they did not respond to a mechanical stimulus. The experiment was performed three times. Mean fraction alive indicates the average survival among the multiple trials and the error bar represents the standard deviation. *P*-value was calculated using Student's *t*-test. Click here for file

Additional file 12 ***hpl-2 *does not influence levels of ceramides and neutral lipids**. **(a) **Ceramides (elution fraction 2) isolated from wild-type and *hpl-2 *animals were loaded on thin layer chromatography plates and run in a chloroform:methanol (50:5) solvent system. **(b) **Neutral lipids (elution fraction 1 and 3) were deposited on thin layer chromatography plates and run in hexane:diisopropylether:acetic acid (80:20:1). Click here for file
