# Supplementary figures and images for: Caenorhabditis elegans Heterochromatin protein 1 (HPL-2) links developmental plasticity, longevity and lipid metabolism
Source: Genome Biol. 2011 Dec 20;12(12):R123. doi: 10.1186/gb-2011-12-12-r123 (PMC3334618; doi:10.1186/gb-2011-12-12-r123)

wt

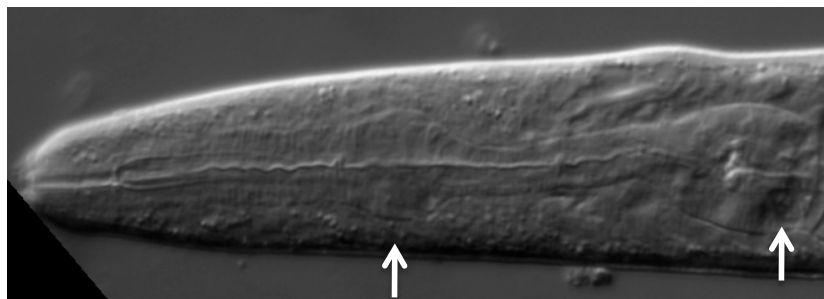

*lin-13::GFP*

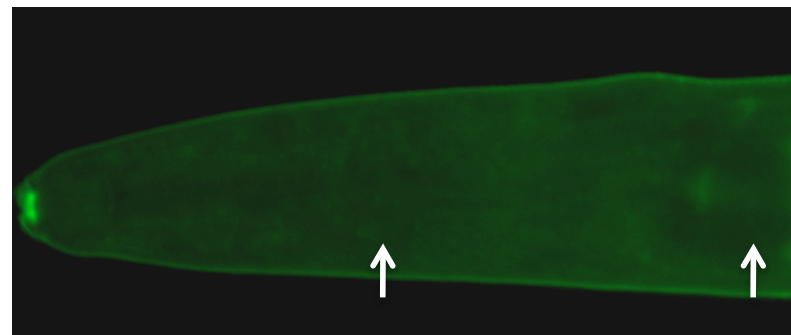

*hpl-2*

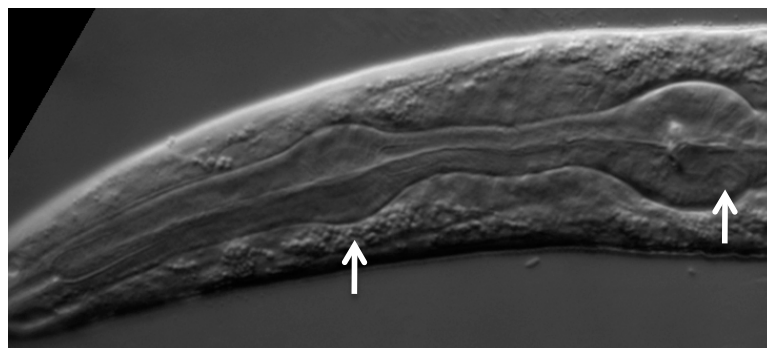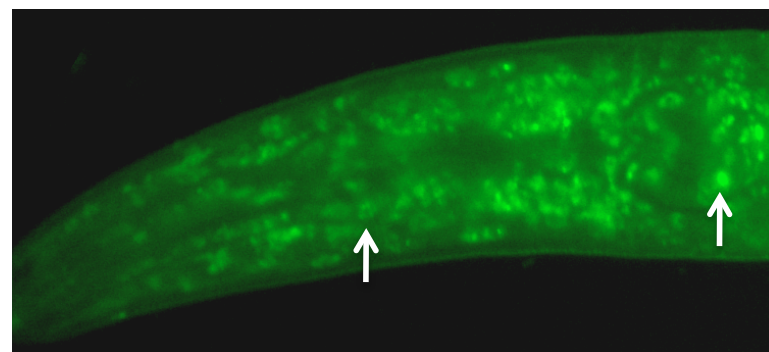

Supplement: Additional file 2 — Increased lin-13::GFP expression in the absence of HPL-2 activity. Expression of a lin-13::GFP translational fusion [108] in wild-type and hpl-2 mutant animals. In hpl-2 mutant animals GFP expression was particularly strong in nuclei in the head region (arrows). [file gb-2011-12-12-r123-S2.PDF]

Meister, Schott et al., Additional file 3

**A**

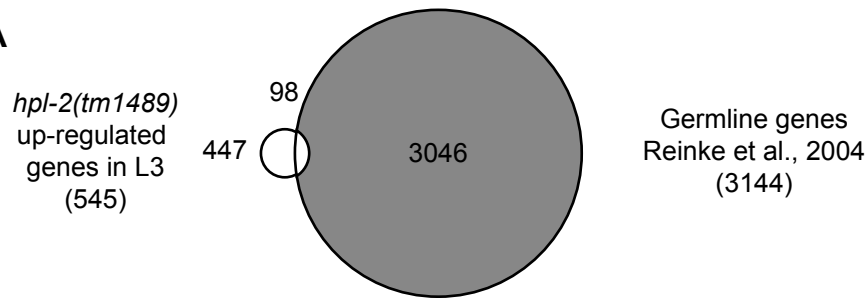

**B**

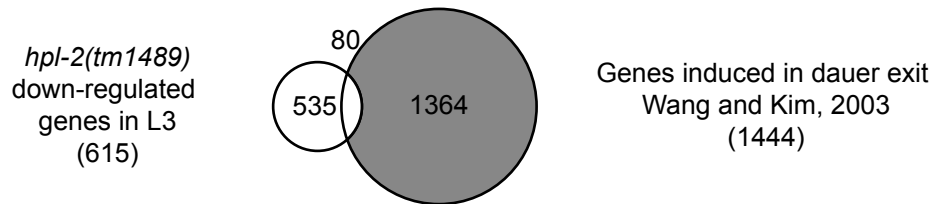

**C**

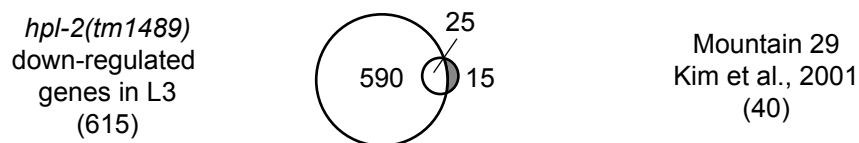

**D**

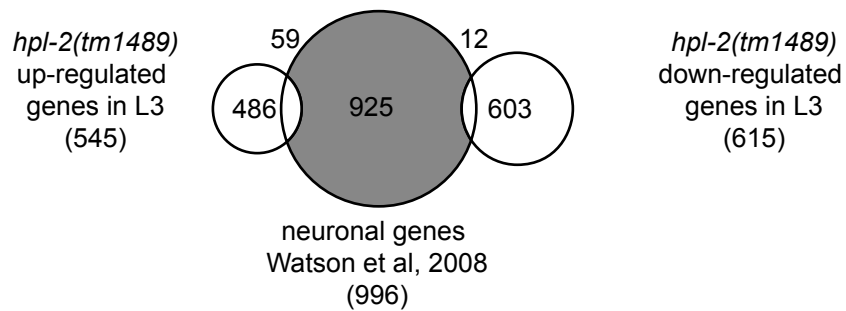

**E**

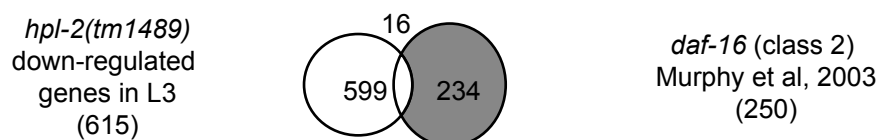

**F**

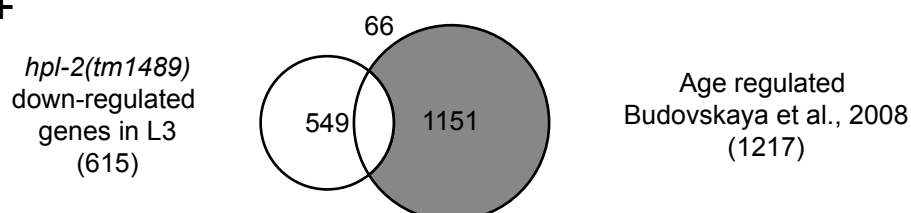

Supplement: Additional file 3 — HPL-2 regulation of specific gene classes. (a-f) Venn diagrams showing overlap between: (a) genes up-regulated in L3 stage hpl-2 mutants and germline genes; (b) genes down-regulated in L3 stage hpl-2 mutants and genes induced in dauer exit; (c) genes down-regulated in L3 stage hpl-2 mutants and genes clustered in expression mountain 29 of the C. elegans three-dimensional topographical expression map; (d) genes regulated in L3 stage hpl-2 mutants and neuronally expressed genes; (e) genes down-regulated in L3 stage hpl-2 mutants and daf-16 target genes; (f) genes down-regulated in L3 stage hpl-2 mutants and aging regulated genes. [file gb-2011-12-12-r123-S3.PDF]

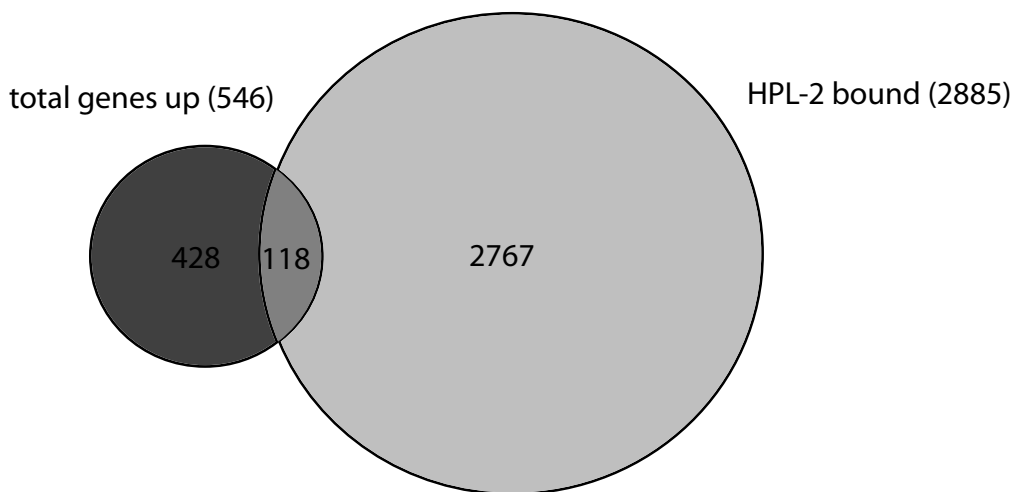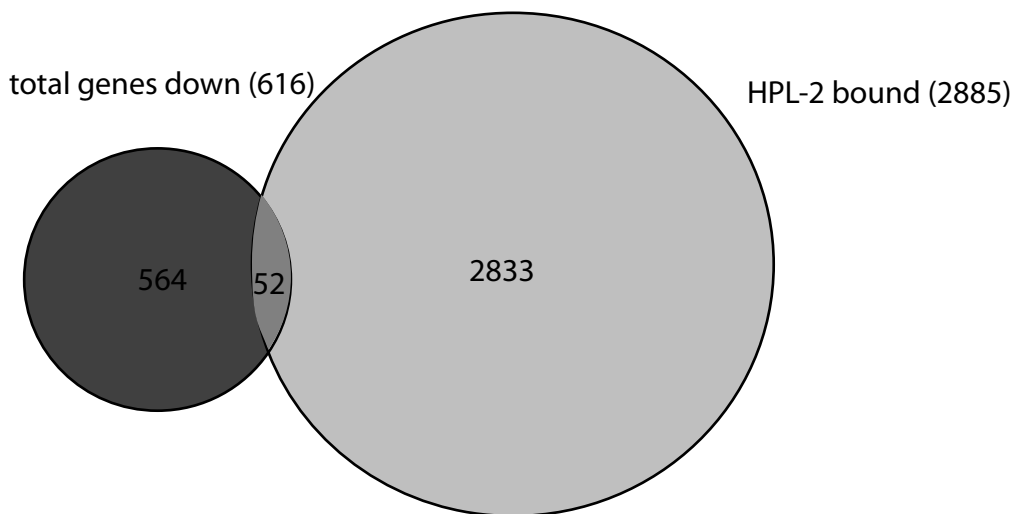

Supplement: Additional file 4 — Overlap between hpl-2-regulated genes and genes bound by HPL-2. Venn diagram showing overlap between genes regulated in L3 stage hpl-2 mutants and genes bound by HPL-2 [29]. [file gb-2011-12-12-r123-S4.PDF]

A.

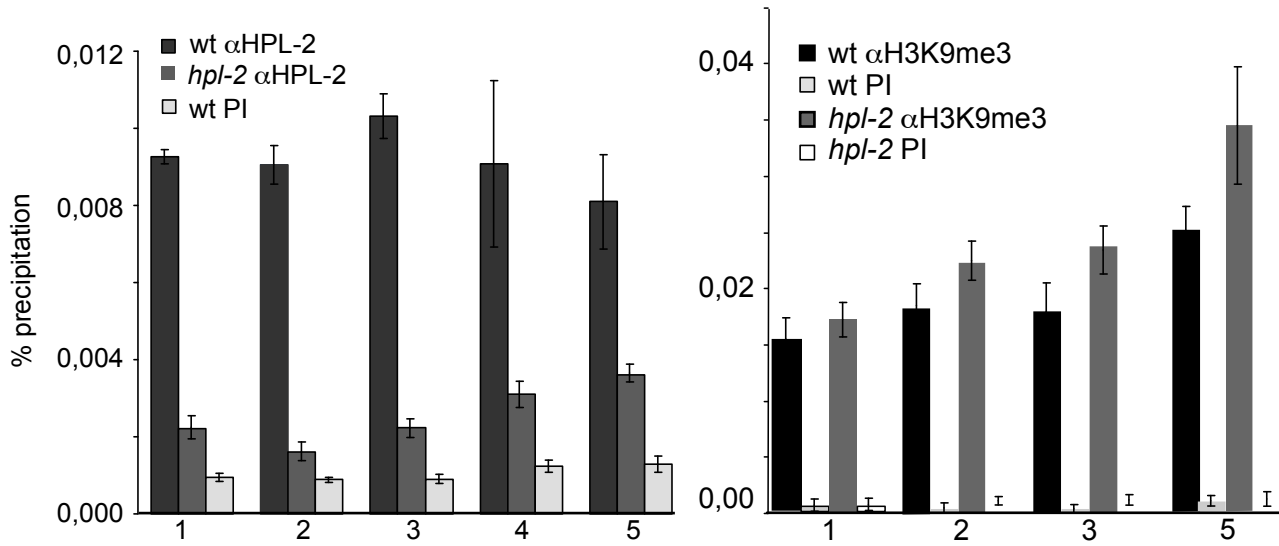

B.

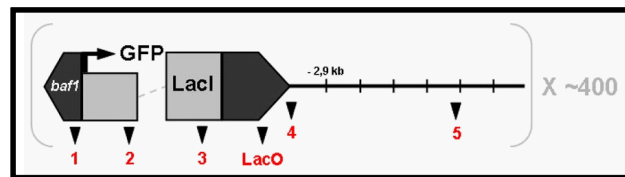

Supplement: Additional file 5 — HPL-2 and H3K9me3 are enriched on repetitive transgenes. (a) Histogram representing the percentage of immunoprecipitation, calculated as the ratio between signal from the antibodies and signal from pre-immune serum, from ChIP experiments along a repetitive transgene. Synchronized populations of L3 wild-type or hpl-2 mutant worms carrying the trangene were subjected to immunoprecipitation using anti-HPL-2, anti-H3K9me3, or pre-immune serum as control. (b) Schematic representation of the transgene used in these experiments [109] showing the position of the primers used for qPCR analysis. Similar results were obtained for three to four independent experiments. [file gb-2011-12-12-r123-S5.PDF]

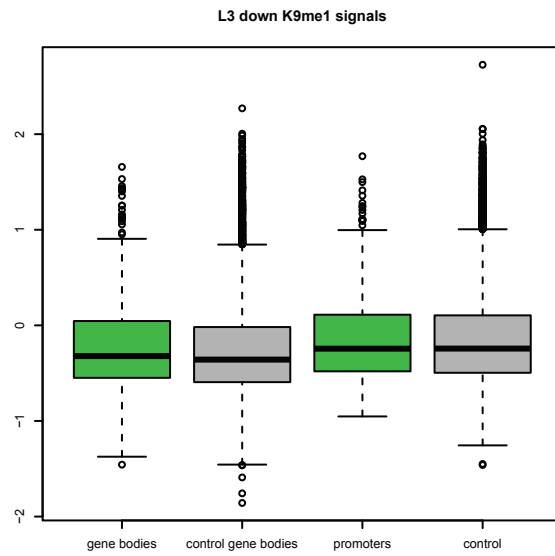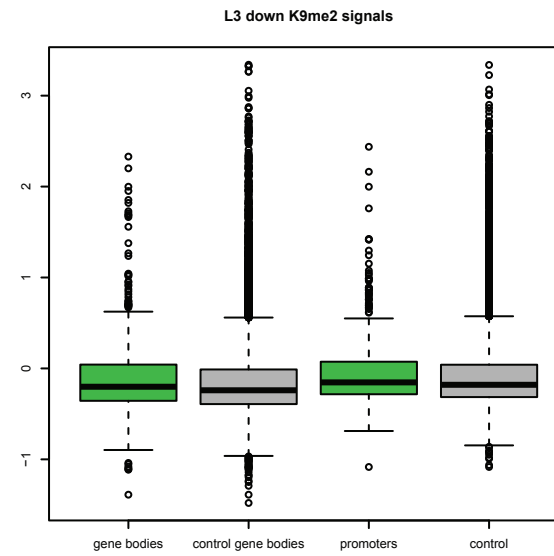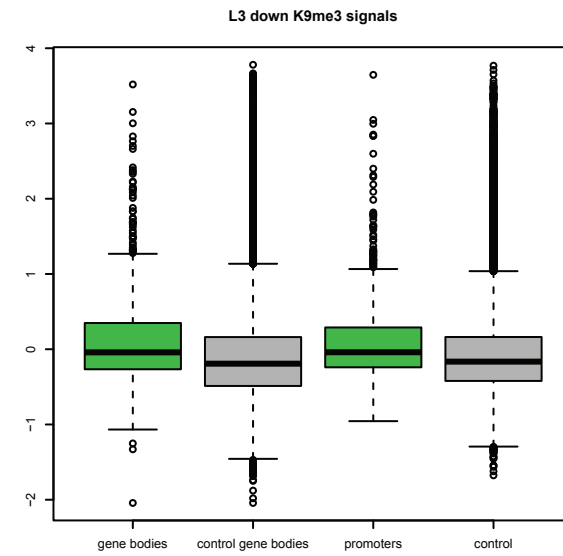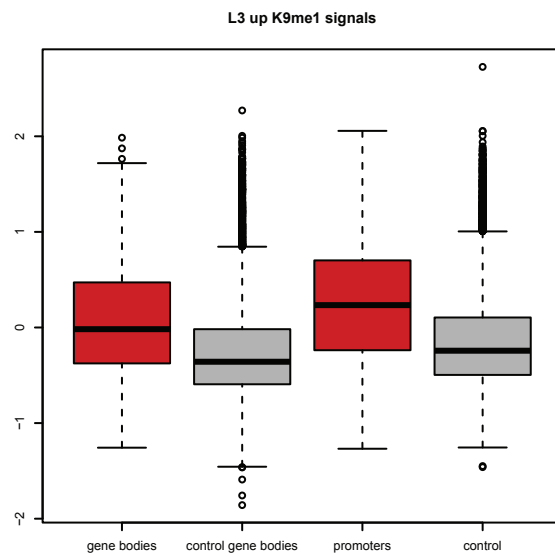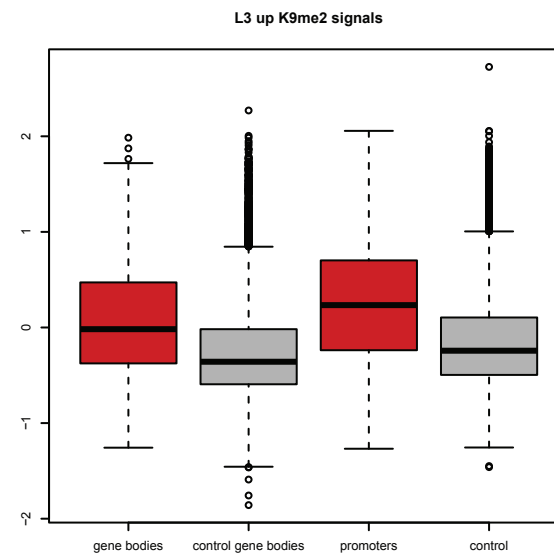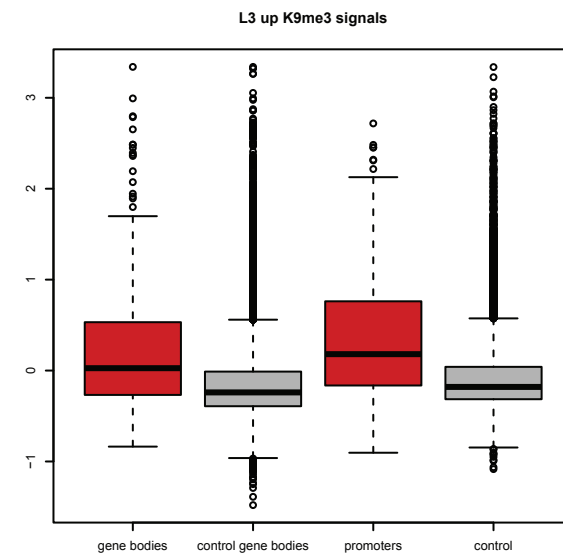

Supplement: Additional file 6 — Repressive histone mark levels on promoters and genes misregulated in hpl-2 mutant worms. For each gene or gene promoter, H3K9 mono-, di- and tri-methyl levels were calculated as the median level observed on the DNA stretch using data provided by the modENCODE Consortium [29]. The criterion for choosing control genes was similar expression levels. Data are presented as box-whisker representation where the bar indicates the median of all up- or down-regulated genes and the upper and lower edges of the box represent the 25th and 75th percentiles, respectively. Up-regulated genes show a significantly higher level of mono-, di- and tri-methylated H3K9, but no enrichment in H3K27 trimethylation, compared to expression-matched control genes. Down-regulated genes show a similar level of mono-, di- and tri-methylated H3K9 compared to expression-matched control genes. [file gb-2011-12-12-r123-S6.PDF]

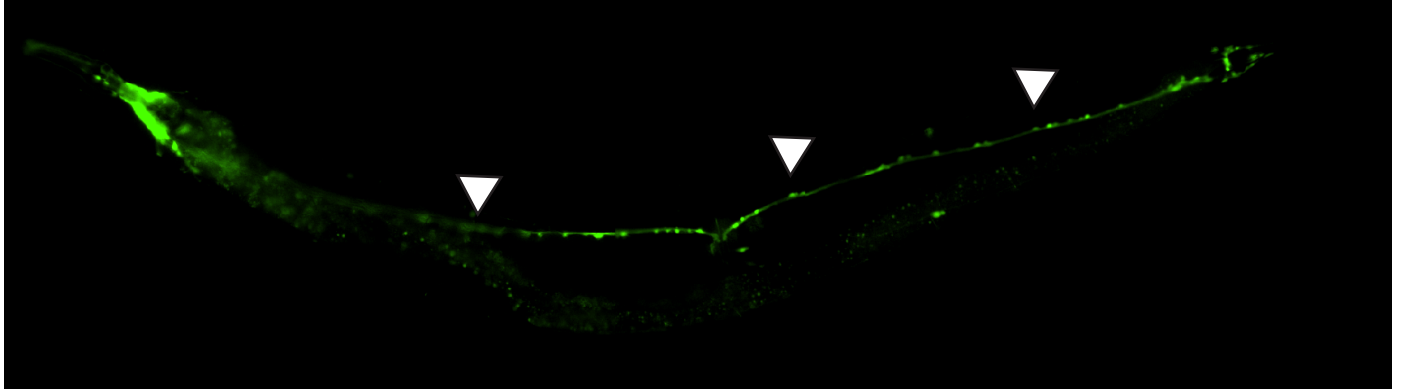

Supplement: Additional file 7 — rab-3p::hpl-2cDNA::GFP transgene is expressed uniquely in neuronal cells. GFP fluorescence is detected in the synaptic-rich regions of the nervous system, including the nerve ring, ventral nerve cord, and dorsal nerve cord (arrowheads). [file gb-2011-12-12-r123-S7.PDF]

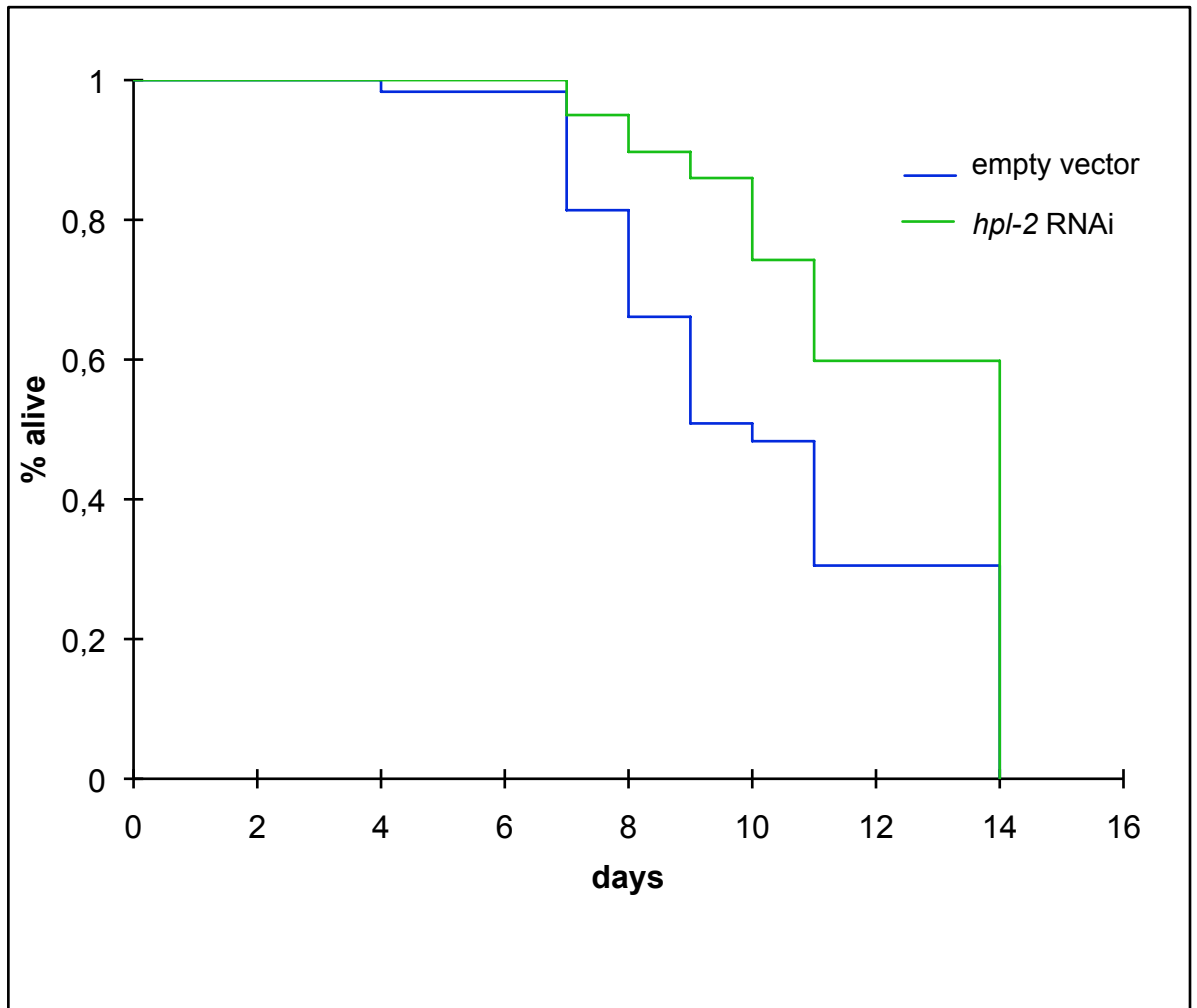

Supplement: Additional file 8 — Increased lifespan of hpl-2(RNAi) animals. Animals at the L4 stage were placed on RNAi or control bacteria at 20°C and allowed to lay eggs. When F1 progeny reached the L4 stage, they were transferred to 25°C for lifespan assays on plates containing 10 μM 5FU. Worms were then transferred to fresh RNAi plates every week. hpl-2 RNAi increased average lifespan by 18% (average lifespans: wild type fed control bacteria, 10.3 ± 0.42 (n = 60); wild type fed hpl-2 RNAi clone 1, 12.2 ± 0.32 days (n = 60; control comparison P = 10-3). Similar results were obtained in two independent tests. [file gb-2011-12-12-r123-S8.PDF]

**Meister, Schott et al., Additional file 9**

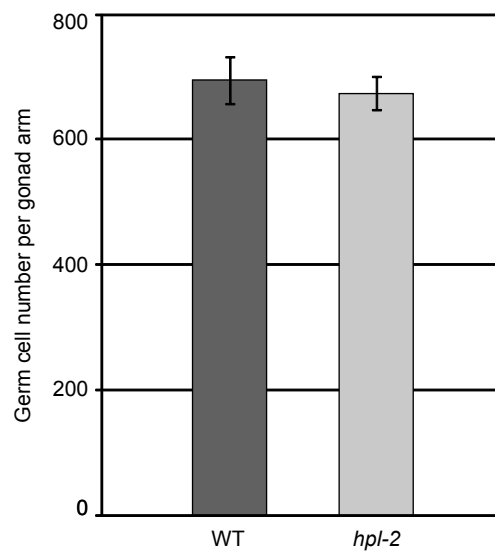

Supplement: Additional file 9 — hpl-2 deletion does not affect the total number of germ cells produced. Data are displayed as the mean (± standard deviation) number of germ cells per gonad arm (n = 4). Germ cell number was obtained by counting germ cell nuclei stained with DAPI in dissected gonads of young adults. [file gb-2011-12-12-r123-S9.PDF]

*daf-16p::daf-16::GFP, 35°C*

WT

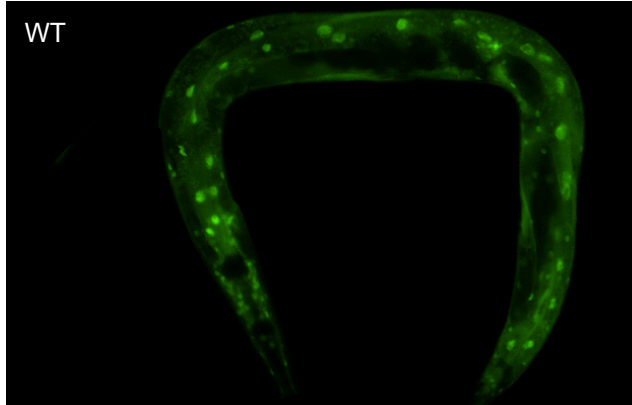

*hpl-2(tm1489)*

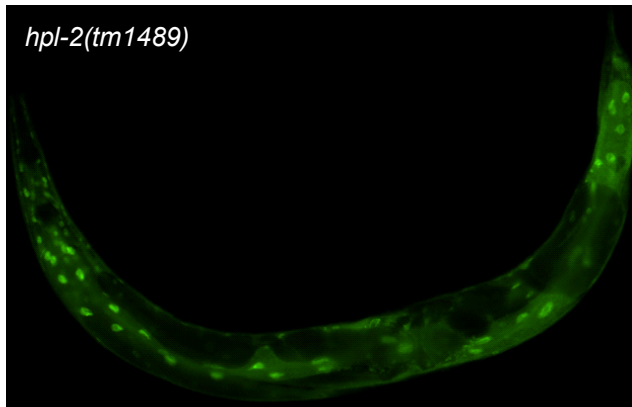

Supplement: Additional file 10 — hpl-2 does not influence DAF-16::GFP localization. Adults carrying a daf-16::gfp transgene (zIs356) were placed on NGM plates at 37°C. After 45 minutes, worms were mounted onto a slide in M9 buffer. Nuclear translocation of DAF-16::GFP was visualized with a fluorescence microscope AxioImager Z1 (Zeiss) equipped with a CoolSnap HQ camera and driven by Metamorph software (Molecular Devices, France). [file gb-2011-12-12-r123-S10.PDF]

Meister, Schott et al., Additional file 11

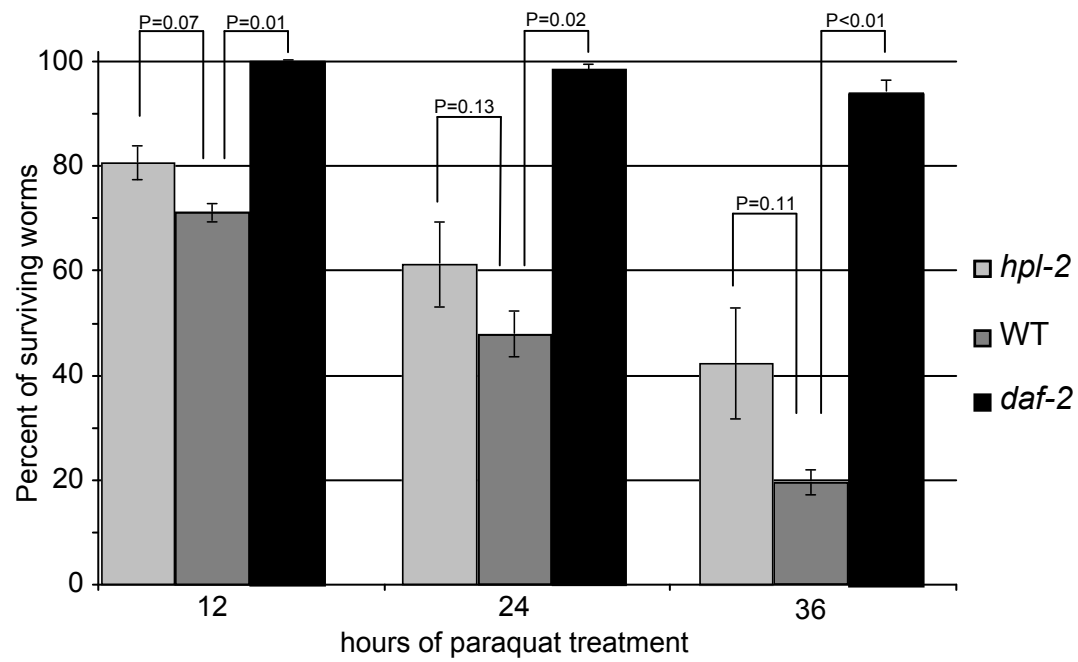

Supplement: Additional file 11 — The hpl-2(tm1489) deletion mutant does not affect resistance to oxidative stress. Adult hermaphrodites were incubated in M9 buffer with 100 mM paraquat. After incubation at 20°C for the specified duration, survival was measured. Worms were scored as dead when they did not respond to a mechanical stimulus. The experiment was performed three times. Mean fraction alive indicates the average survival among the multiple trials and the error bar represents the standard deviation. P-value was calculated using Student's t-test. [file gb-2011-12-12-r123-S11.PDF]

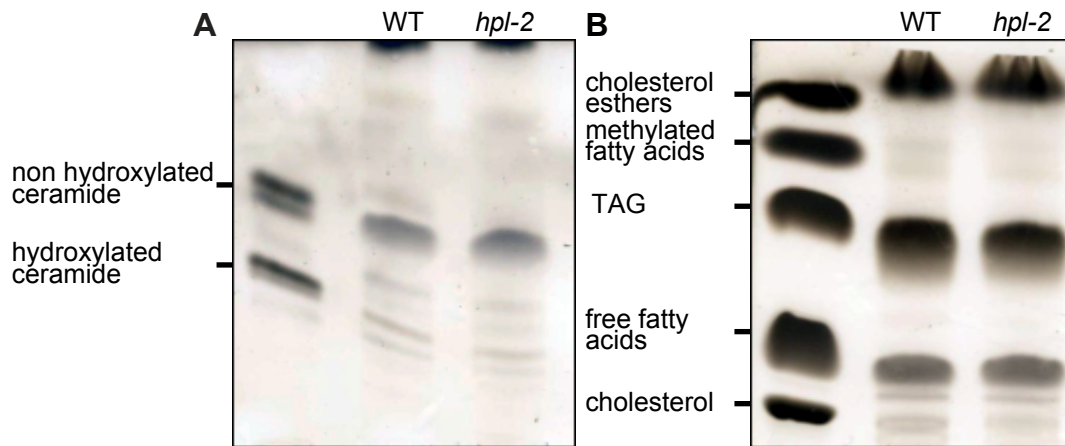

Supplement: Additional file 12 — hpl-2 does not influence levels of ceramides and neutral lipids. (a) Ceramides (elution fraction 2) isolated from wild-type and hpl-2 animals were loaded on thin layer chromatography plates and run in a chloroform:methanol (50:5) solvent system. (b) Neutral lipids (elution fraction 1 and 3) were deposited on thin layer chromatography plates and run in hexane:diisopropylether:acetic acid (80:20:1). [file gb-2011-12-12-r123-S12.PDF]
